# ZFP92, a KRAB domain zinc finger protein enriched in pancreatic islets, binds to B1/Alu SINE transposable elements and regulates retroelements and genes

**DOI:** 10.1371/journal.pgen.1010729

**Published:** 2023-05-08

**Authors:** Anna B. Osipovich, Karrie D. Dudek, Linh T. Trinh, Lily H. Kim, Shristi Shrestha, Jean-Philippe Cartailler, Mark A. Magnuson

**Affiliations:** 1 Department of Molecular Physiology and Biophysics, Vanderbilt University, Nashville, Tennessee, United States of America; 2 Center for Stem Cell Biology, Vanderbilt University, Nashville, Tennessee, United States of America; 3 Department of Cell and Developmental Biology, Vanderbilt University, Nashville, Tennessee, United States of America; 4 College of Arts and Sciences, Vanderbilt University, Nashville, Tennessee, United States of America; University of Colorado Boulder, UNITED STATES

## Abstract

Repressive KRAB domain-containing zinc-finger proteins (KRAB-ZFPs) are abundant in mammalian genomes and contribute both to the silencing of transposable elements (TEs) and to the regulation of developmental stage- and cell type-specific gene expression. Here we describe studies of zinc finger protein 92 (*Zfp92*), an X-linked KRAB-ZFP that is highly expressed in pancreatic islets of adult mice, by analyzing global *Zfp92* knockout (KO) mice. Physiological, transcriptomic and genome-wide chromatin binding studies indicate that the principal function of ZFP92 in mice is to bind to and suppress the activity of B1/Alu type of SINE elements and modulate the activity of surrounding genomic entities. Deletion of *Zfp92* leads to changes in expression of select LINE and LTR retroelements and genes located in the vicinity of ZFP92-bound chromatin. The absence of *Zfp92* leads to altered expression of specific genes in islets, adipose and muscle that result in modest sex-specific alterations in blood glucose homeostasis, body mass and fat accumulation. In islets, *Zfp92* influences blood glucose concentration in postnatal mice via transcriptional effects on *Mafb*, whereas in adipose and muscle, it regulates *Acacb*, a rate-limiting enzyme in fatty acid metabolism. In the absence of *Zfp92*, a novel TE-*Capn11* fusion transcript is overexpressed in islets and several other tissues due to de-repression of an IAPez TE adjacent to ZFP92-bound SINE elements in intron 3 of the *Capn11* gene. Together, these studies show that ZFP92 functions both to repress specific TEs and to regulate the transcription of specific genes in discrete tissues.

## Author summary

Nearly half of the genomic DNA in mammals is comprised of transposable genetic elements (TEs) that provide a massive source of sequence diversity and regulatory activity. Most TEs are silenced by host factors that include KRAB domain-containing zinc-finger proteins (ZFPs), the majority of which remain uncharacterized. In this study, we analyzed mice that lack *Zfp92*, a mammalian conserved KRAB-ZFP that is highly expressed in pancreatic islets. We found that *Zfp92* principally binds to short interspersed nuclear elements (SINEs) and modulates the activity of these and other TEs and nearby genes. *Zfp92* positively regulates the expression of *Mafb*, a transcription factor important for normal islet development and function, and *Acacb*, an enzyme important for lipolysis in adipose and muscle tissues. The absence of *Zfp92* causes the re-activation of an IAPez TE in intron 3 of the *Capn11* gene, resulting in the marked overexpression of a novel TE-*Capn11* fusion transcript in islets and several other tissues. These findings provide another example of how TEs and their partnering KRAB-ZFPs may affect the regulation and function of nearby genes, thereby contributing in important ways to cellular development and function.

## Introduction

Zinc finger proteins (ZFPs) containing classical C_2_H_2_ (Cys_2_-His_2_) zinc finger domains, a conserved DNA-binding motif, represent a large but poorly explored family of higher eukaryotic transcription factors (TFs) [1]. The human genome encodes 747 C_2_H_2_ finger-containing proteins which represent nearly half of all human TFs [2], most of which bind DNA and regulate transcription [3]. Each C_2_H_2_ zinc finger consists of a 23–30 amino-acid motif stabilized by a zinc ion and binds to a 3–4 nucleotide target DNA sequence [4]. ZFPs typically contain one to more than a dozen of zinc finger motifs as well as other domains such as protein-protein interacting SCAN domains (7% of C_2_H_2_ ZFPs), transcriptional regulatory BTB/POZ domains (7%), and KRAB domains (43%) [5,6]. Some C_2_H_2_-ZFPs have been highly conserved throughout evolution and partner with conserved nuclear receptors, consistent with their involvement in preserved biological activities such as general transcriptional regulation and early organogenesis [7]. However, other C_2_H_2_-ZFPs, particularly those containing repressive KRAB domains, have undergone evolutionary gene duplication and expansion, particularly in mammals, and are thought to be involved in modulating complex species-specific gene transcriptional networks [7].

KRAB-ZFPs contain an N-terminal Krüppel-associated box (KRAB) domain that functions by attracting repressive co-factors, such as the principal co-repressor KRAB-associated protein-1 (KAP1, also known as TRIM28), heterochromatin protein 1 (HP1), histone deacetylases, corepressor complexes (NuRD and NCOR), the histone methyltransferase SETDB1 and DNA methyltransferases [8]. Despite their genomic abundance, the function of most KRAB-ZFPs remains undefined [9]. While KRAB-mediated transcriptional repression is linked to diverse biological functions including regulation of cell proliferation, differentiation and apoptosis [10], evidence also points to a primary role of KRAB-ZFPs in silencing of transposable elements (TEs) in the genome [11–13].

TEs account for more than half of the human and murine genomes [14] and are important drivers of genomic regulatory diversity and evolution. However, due to their abundance, they also represent threats to genomic integrity and gene regulation [15,16]. Retrotransposons, the predominant TEs found in mammalian genomes, have been grouped into three classes: short interspersed nuclear elements (SINEs), long interspersed nuclear elements (LINEs), and long terminal repeat retrotransposons (LTRs) [17]. The LTR class is represented by endogenous retroviruses (ERVs) containing retroviral structures with gag-pol-env genes flanked by LTRs that contain cis-regulatory elements [18]. While most retroelements are inactive due to silencing and mutations, some remain or can become active and may contribute to disease through deleterious insertion mutations and dysregulation of host gene expression [19,20].

Many KRAB-ZFPs have similar evolutionary ages to their target TEs, and exist in roughly equal proportions, suggesting that they co-evolved in response to TE infiltration [21]. Indeed, a dynamic competition, or so-called arms race, between TEs and KRAB-ZFPs has been suggested to have occurred during mammalian evolution with the selection, expansion and adaptation of specific KRAB-ZFP serving to silence TE expression and retrotransposon activity [22,23]. However, another proposed complementary evolutionary driver is the domestication of TE-derived regulatory sequences whereby KRAB-ZFPs co-evolve with TEs to rewire gene regulatory networks in each species, therefore impacting on development and function of different tissues [9,24]. For example, *Zfp932* and *Gm15446*, two mouse KRAB-ZFPs, have been shown to not only repress ERVs but to also modify the expression of neighboring genes in embryonic stem cells and adult tissues [25]. Similarly, two primate-specific KRAB-ZFPs, *ZNF417* and *ZNF587*, repress human ERV and SINE elements in embryonic stem cells and, through expression in specific brain regions, influence the differentiation and neurotransmission profile of neurons [26]. Indeed, both TEs and their KRAB-ZFP controllers have been shown to regulate gene expression in the human brain in a region-specific manner and to drive transcriptional innovation [27,28]. These studies suggest that TEs and KRAB-ZFPs establish transcriptional networks that regulate cell differentiation and function in a species- and tissue-specific manner.

Pancreatic endocrine cells in the islets of Langerhans regulate blood glucose homeostasis through secretion of hormones into the bloodstream. There are five distinct cell types, the most abundant of which are α- and β- cells that secrete glucagon and insulin, respectively [29]. Alterations in TFs that regulate differentiation and function of β-cells contribute to development of diabetes [30]. Previously, we derived a developmental gene co-expression network (GCN) for mouse pancreatic β-cells that revealed 81 C_2_H_2_-ZFPs whose expression was associated with endocrine specification and pancreatic β-cell maturation [31]. Among these genes, *Zfp92*, a KRAB-ZFP located on the X chromosome, was found to be highly expressed in nascent and mature β-cells, prompting us to explore its role in pancreatic β-cell gene regulation by using CRISPR/Cas9 to generate a global knockout (KO) allele.

Here, we describe *Zfp92* KO mice. We found that the absence of *Zfp92* in islets modestly impairs insulin gene transcription in post-natal mice via transcriptional effects on *Mafb*. Additionally, the analysis revealed that *Zfp92* KO male mice had impaired expression of *Acacb*, a rate-limiting enzyme in fatty acid β-oxidation, that results in sex-specific alterations in insulin sensitivity and body mass when fed a high fat diet. However, the major role of *Zfp92* is to potentially suppress the activity of B1/Alu SINE elements by binding to a 28 bp consensus ZFP92 binding motif. De-repression of an IAPez ERV located next to SINE elements in *Capn11* gene intron in the absence of *Zfp92* leads to marked overproduction of a novel fusion TE-*Capn11* transcript the function of which remains unknown. Together, our data reveal a multifunctional role for *Zfp92* in regulating gene expression in the mouse, providing another example of how KRAB-ZFPs can simultaneously be involved in suppressing specific TEs and in the regulation of tissue-specific gene regulation.

## Results

### *Zfp*92 tissue expression, mRNA forms and *Zfp*92 knockout mice

**Fig 1:**
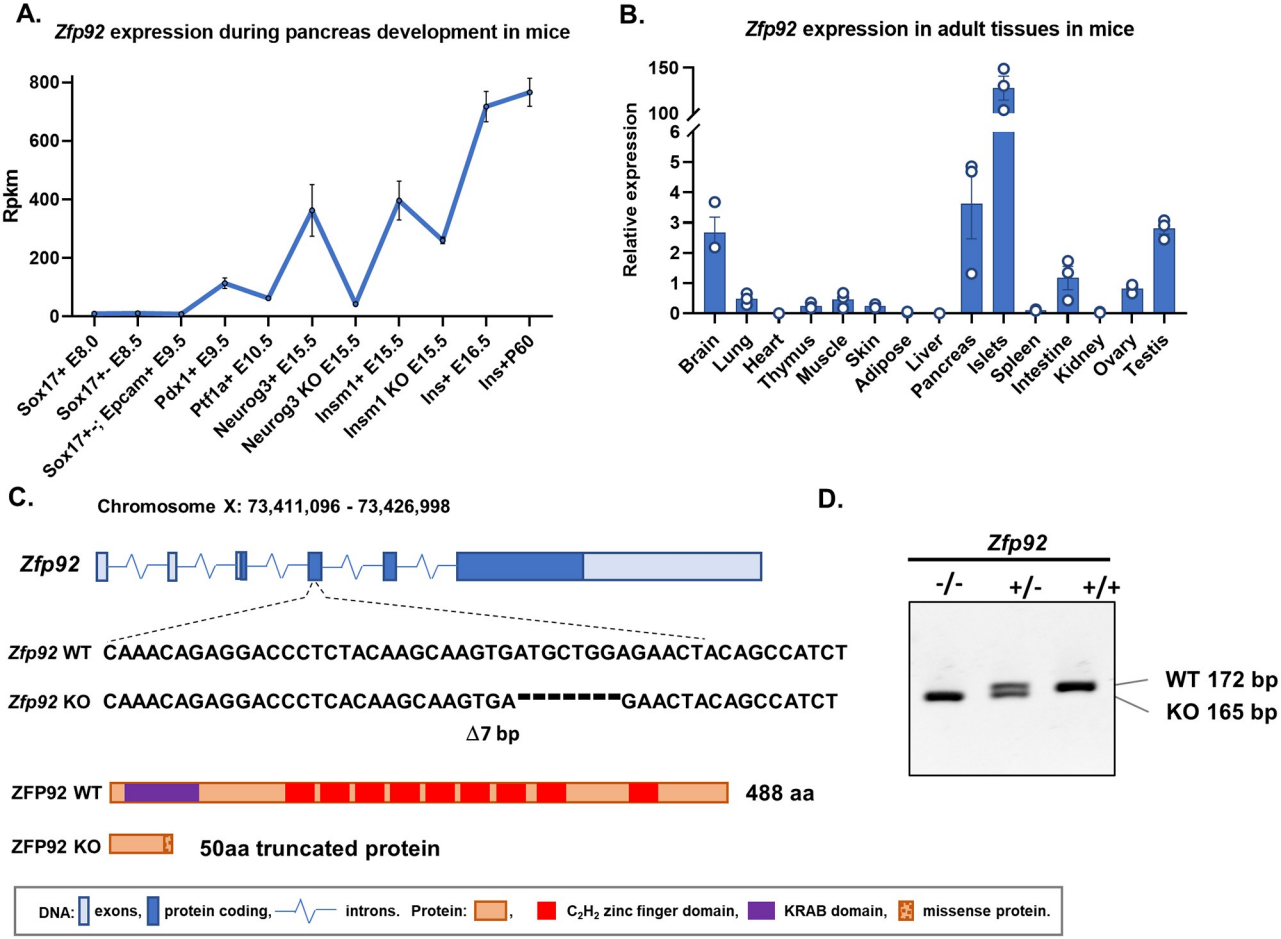
*Zfp92* expression and CRISPR-derived knock-out allele. **A**) Expression levels of *Zfp92* mRNA profiled by RNA-seq in sorted pancreatic developmental lineages: gut tube endoderm (Sox17^+^ at E8.0/8.5), posterior foregut endoderm (*Pdx1*^*+*^ at E9.5), pancreatic multipotent progenitor cells (*Ptf1a*^*+*^ at E10.5), endocrine progenitor cells (*Neurog3*^*+*^ and *Insm1*^*+*^ at E15.5), nascent β-cells (Ins^+^ at E16.5), and adult β-cells (Ins^+^ at P60). Two profiled mutant conditions for endocrine progenitor cells (*Neurog3*^*-/-*^ and *Insm1*^*-/-*^ at E15.5) indicate that *Zfp92* is reduced in the absence of *Neurog3*. N = 3.**B**) Tissue expression patterns of *Zfp92* determined by the RT-qPCR analysis of RNA from tissues from adult mice (10 weeks old). All tissues, except ovaries, are from male mice. N = 3. Relative expression is calculated in comparison to the same gene expression in the intestine. Br, brain; Lu, lung; He, heart; Th, thymus; Mu, skeletal muscle; Sk, skin; Ad, adipose; Li, liver; Pa, whole pancreas; Is, pancreatic islets; Sp, spleen; In, small intestine; Ki, kidney; Ov, ovary; Te, testis. **C**) Schematic representation of *Zfp92* gene and relative gene location and sequence of CRISPR/Cas9 generated 7 bp deletion. Deletion of 7 bp in *Zfp92* causes a frameshift that interrupts ZFP92 protein translation resulting in a predicted 50 aa peptide containing 8 missense amino acids. **D**) Representative image of a PCR genotyping gel for *Zfp92* wild type (WT), heterozygous (Het), and knockout (KO) mice.

Using data from our prior gene correlation network analysis of purified cell populations representing main stages of β-cell development from embryonic day (E) 8.0 to postnatal day (P) 60 in mice [31], we observed that *Zfp92* expression is increased at E15.5 in pro-endocrine pancreatic progenitor cells that express *Neurog3*, further upregulated in nascent β-cells and maintained in mature β-cells (Fig 1A). RT-qPCR of mouse adult tissues showed that *Zfp92* is predominantly expressed in pancreatic islets, with higher levels also detected in brain, whole pancreas, and testis samples (Fig 1B). In addition, scRNA-Seq data from *Tabula Muris* [32] indicate that *Zfp92* is predominantly expressed in β-cells, with lower levels in α- and δ-cells in mouse islets [31].

Since the *Zfp92* gene model predicts two mRNA forms, a long form that encodes a full length ZFP92 protein and a short form that encodes a C-terminally truncated ZFP92 (S1A Fig), we designed primer pairs specific to each form and performed RT-PCR and qPCR on islet samples to establish which is predominantly expressed in islets, (S1A–S1D Fig). The results show that the predominant RNA form expressed in pancreatic islets is the longer variant that encodes a 488 aa protein containing an N-terminal KRAB domain and an array of 8 C_2_H_2_ zinc fingers.

Since *Zfp92* is highly enriched in both islets and pancreatic β-cells, we hypothesized that this gene may serve an important but undefined role in pancreatic islet development and/or function. For this reason, we performed CRISPR/Cas9 mutagenesis by injecting guide RNAs targeting *Zfp92* into mouse zygotes that express CRISPR/Cas9. Analysis of the offspring revealed a founder animal bearing a 7 bp frameshift deletion downstream of the translation initiation codon in the gene (Fig 1C and 1D). The 7 bp deletion creates a frameshift in an open reading frame that leads to a premature STOP codon resulting in a truncated 50 aa protein thereby generating a null (KO) allele for *Zfp92* (Fig 1C).

### *Zfp*92 KO mice exhibit sex-specific alterations in body weight and fasting blood glucose

**Fig 2:**
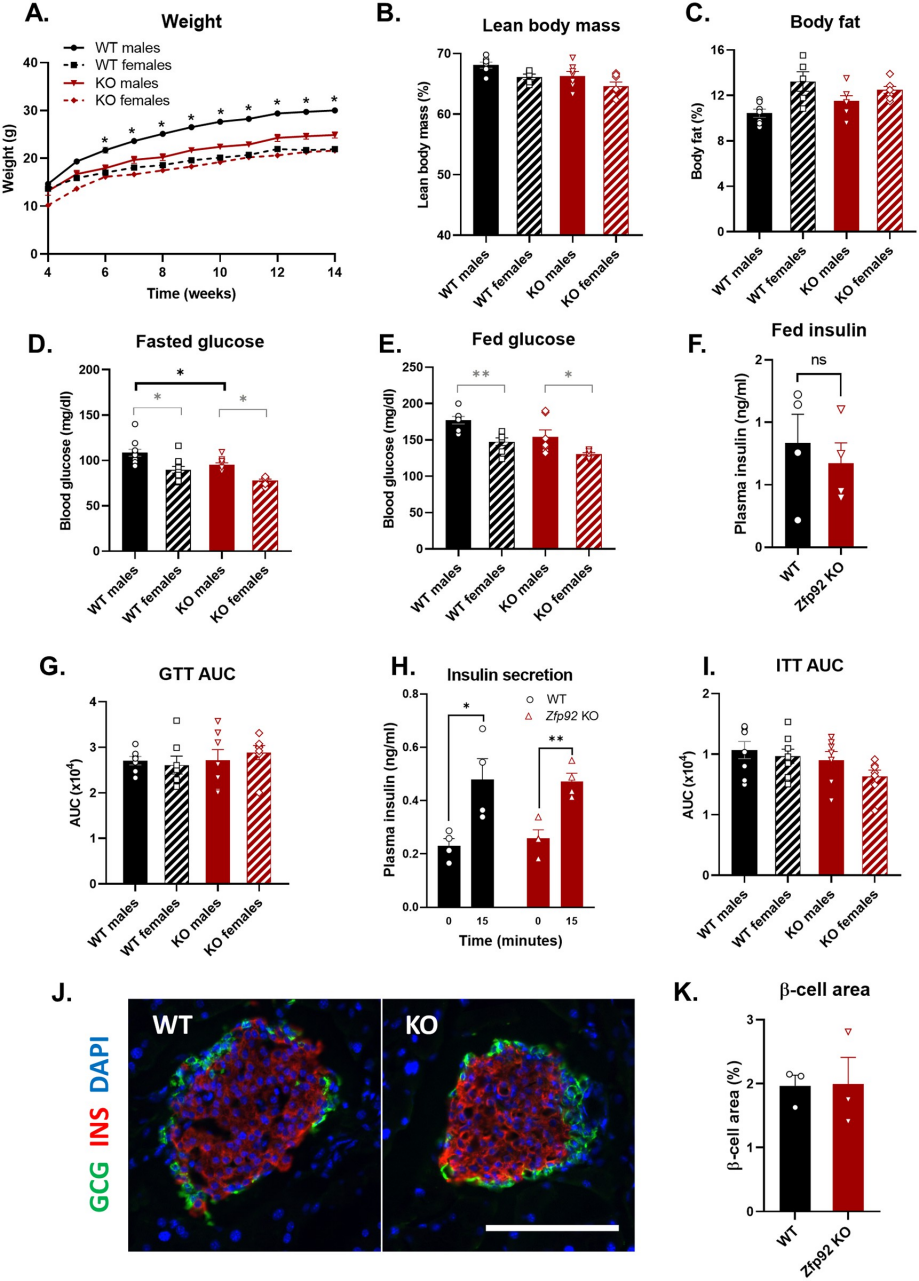
Growth, body fat composition, glucose homeostasis, and islet structure of *Zfp92* KO mice. **A**) Body weight measurements from 4 to 14 weeks showing *Zfp92* KO male mice have reduced growth in comparison to WT males (n = 7,8 for each sex and genotype. Lean body mass (**B**) and body fat (**C**) measurements show no difference between KO and WT mice body composition at (n = 5–7 for each sex and genotype). Fasted (**D**) and fed (**E**) blood glucose concentration measurements. KO mice have lower fasting blood glucose than WT mice. (n = 8–11 for each sex and genotype). **F**) Random fed plasma insulin levels (n = 4, male mice). **G**) Intraperitoneal glucose tolerance test (GTT) results are presented as the area under the curve (AUC) measurements for S2A Fig (n = 7 for each sex and genotype). **H**) Insulin secretion in response to glucose injection in GTT (n = 4, males). Both genotypes show an equal increase in plasma insulin concentration 15 min after injection. **I**) Intraperitoneal insulin tolerance test (ITT) results presented as the area under the curve (AUC) measurements for S2B Fig (n = 7–8 for each sex and genotype). **J**) Immunofluorescent staining for insulin (red) and glucagon (green) of pancreatic islet tissues from WT and *Zfp92* KO mice. Nuclei are stained with DAPI (blue). Scale bar: 100 microns. **K**) Quantification of hormone-positive areas in KO and WT islets (n = 3, males at 14–15 weeks). **B-K**) All mice are 14–15 weeks old. Statistical significance between KO and WT samples is indicated by black brackets, and between sexes by gray brackets. Error bars: ± SEM. **p≤0.01; *p≤0.05. p-value determined by ANOVA or by t-test (panels **F, K**).

*Zfp92* homozygous KO mice were viable, fertile and did not demonstrate any gross abnormalities. Weekly monitoring of body weight for 10 weeks of male and female mice maintained on normal diet revealed that *Zfp92* KO males have lower body weight than their wild type (WT) counterparts beginning from 6 months of age (Fig 2A). We did not observe any significant differences in body composition measurements at 14 months indicating that weight differences are not due to disproportionate accumulation of fat (Fig 2B and 2C). We assessed glucose homeostasis in WT and KO mice by measuring fasting and fed blood glucose, plasma insulin, and by performing intraperitoneal glucose and insulin tolerance tests (Fig 2D–2I, S2A and S2B Fig). These measurements revealed a slight decrease in fasting blood glucose in male mice (Fig 2D), however, the mice showed similar fed blood glucose and insulin, insulin secretion in response to glucose injection (Fig 2H), and no differences in glucose (Fig 2G) and insulin tolerance (Fig 2I). Immunostaining and morphometric analyses of pancreatic tissue sections from KO and WT mice did not show any abnormalities in islet architecture, islet composition or β-cell area (Fig 2J and 2K).

### *Zfp*92 KO mice have altered body composition and elevated fasting blood glucose on a high fat diet

**Fig 3:**
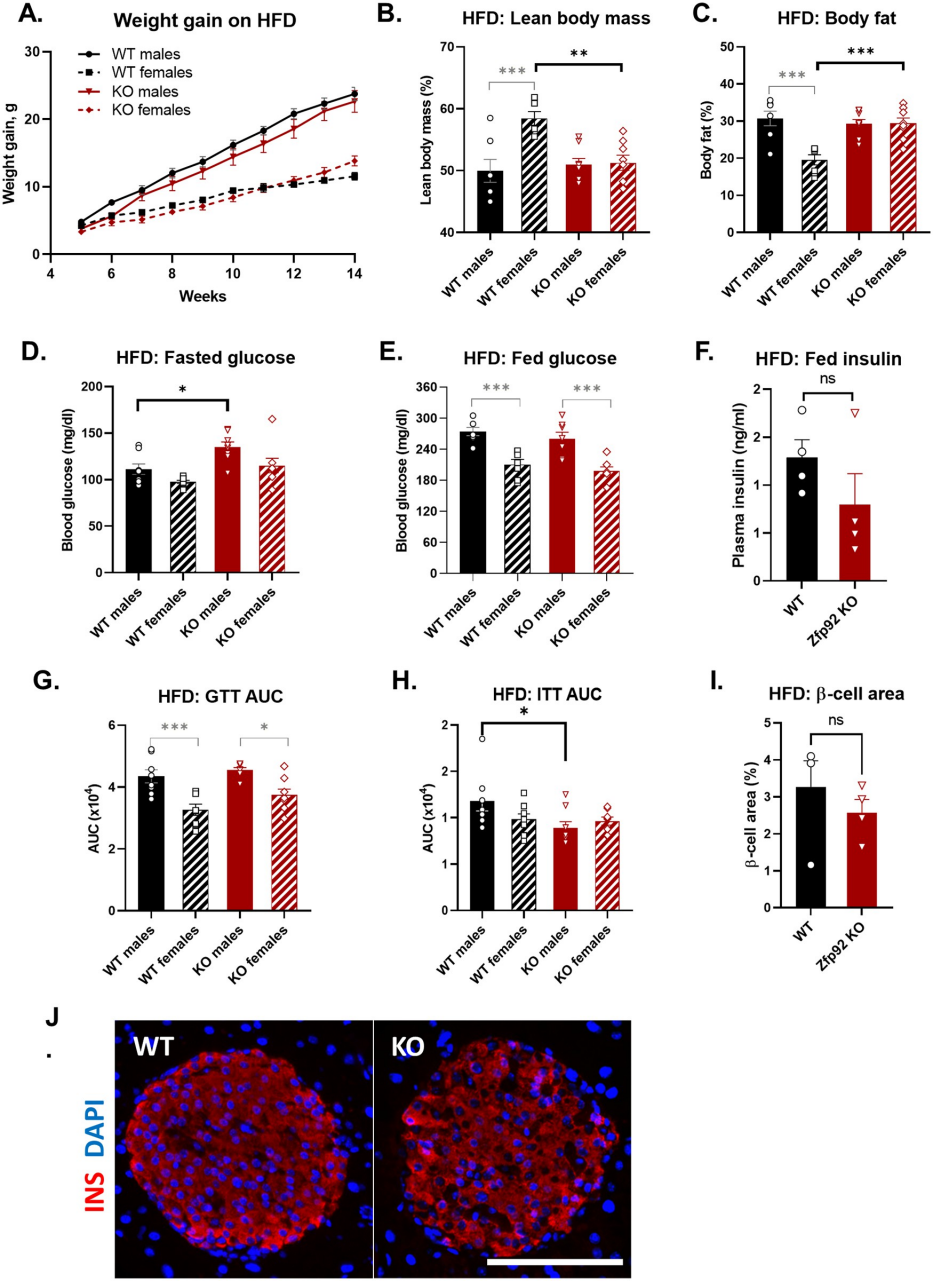
Effects of a high fat diet (HFD) on weight gain, body composition, glucose homeostasis and islet structure of *Zfp92 KO* mice. **A**) Weight gain on HFD for 10 weeks was similar for both wild type (WT) and *Zfp92* knockout (KO) animals (n = 7–9 for each sex and genotype). Lean body mass (**B**) and body fat (**C**) measurements show an increase in body fat content and a decrease in lean body mass in KO female mice on HFD (n = 7–8 for each sex and genotype). Fasted (**D**) and fed (**E**) blood glucose concentration measurements. KO male mice have higher fasting blood glucose than WT mice on HFD (n = 7,8 for each sex and genotype). **F**) Random fed plasma insulin levels (n = 4, male mice). **G**) Intraperitoneal glucose tolerance test (GTT) results are presented as the area under the curve (AUC) measurements for S2C Fig (n = 8 for each sex and genotype). **H**) Intraperitoneal insulin tolerance test (ITT) results are presented as the area under the curve (AUC) measurements for S2D Fig (n = 8 for each sex and genotype). KO male mice have higher insulin sensitivity than WT mice on HFD. **I**) Quantification of insulin-positive β-cell areas in KO and WT islets on HFD (n = 4, males at 14–15 weeks). **J**) Immunofluorescence staining for insulin (red) of pancreatic islet tissues from HFD-fed WT and *Zfp92* KO mice. Nuclei are stained with DAPI (blue). Scale bar: 100 microns. **B-J**) All mice are 14–15 weeks old. Statistical significance between KO and WT samples is indicated by black brackets, and between sexes by gray brackets. Error bars: ± SEM. **p≤0.01; *p≤0.05. p-value determined by ANOVA or by unpaired t-test (panels **F, K**).

To investigate whether *Zfp92* influences β-cell function in response to metabolic stress, we fed WT and *Zfp92* KO mice a high fat diet (HFD) for 10 weeks and then assessed body composition and glucose homeostasis at 14–15 weeks of age. While WT and KO mice gained similar amounts of weight on HFD (Fig 3A), KO female mice had decreased lean body mass and increased body fat after 10 weeks on a HFD (Fig 3B and 3C). Glucose homeostasis measurements showed that KO male mice have slightly increased fasting blood glucose (Fig 3D), but no significant differences in fed blood glucose, insulin, or glucose tolerance were found (Fig 3E–3G, S2C and S2D Fig). Male KO mice had increased insulin sensitivity, suggesting that *Zfp92* affects the function of one or more insulin target tissues (Fig 3H). Feeding of a HFD did not significantly affect β-cell area in mice lacking *Zfp92* (Fig 3I and 3J). Combined, these data indicate that the absence of *Zfp92* has only a mild effect on β-cell function, even during metabolic stress brought on by the feeding of a HFD. However, sex-specific differences in fat accumulation and insulin sensitivity were observed, suggesting a role for *Zfp92* in insulin target tissues such as adipose, muscle and liver.

### Transcriptional profiling of *Zfp*92 KO islets reveals an increase in *Capn11* and a decrease in *Mafb* expression

**Fig 4:**
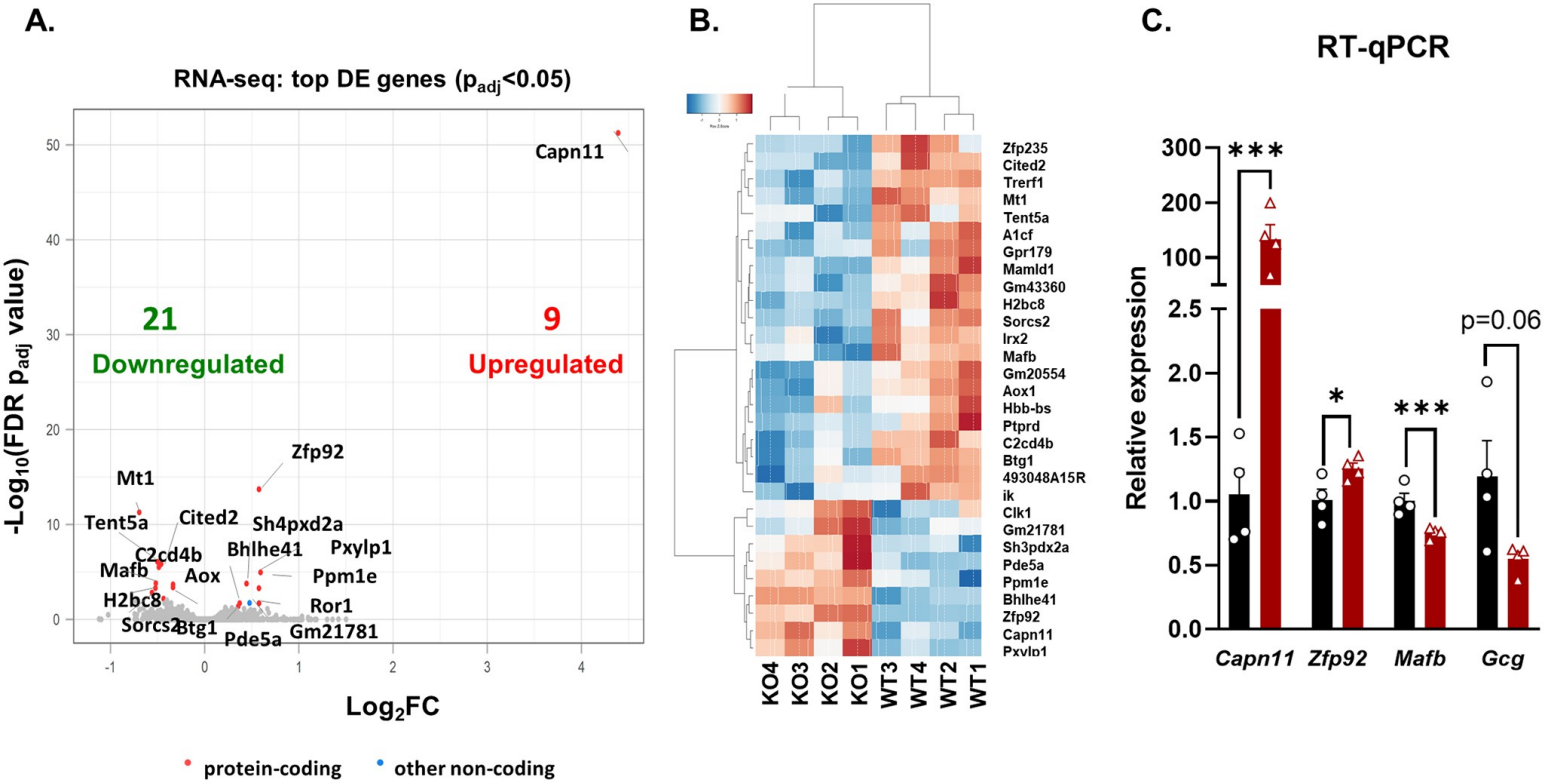
RNA-seq analysis of islets from *Zfp92* KOs shows an increase in *Capn11* and a decrease in *Mafb* gene expression. **A**) Volcano plot showing differentially expressed genes (Log_2_Fold Change (FC) over p_adj_-value) in *Zfp92* KO vs WT islets from 15 week-old male mice (N = 4). The top 10 differentially expressed genes (based on p_adj-_value) are indicated by names and total numbers of downregulated (green) and upregulated (red) genes are provided (p_adj_<0.05). **B**) Heat map showing clustering of the top variant genes (based on p-value) dysregulated in *Zfp92* KO vs WT islets. **C**. RT-qPCR analysis of RNA expression for select genes in islet RNA samples collected from 15 week-old male mice. *Zfp92* wild type (WT), and knockout (KO) samples. N = 4, error bars: ± SEM. ***p≤0.001; *p≤0.05. p-value is determined by an unpaired t-test.

To determine whether *Zfp92* influences gene expression in mouse islets, we performed RNA-Seq of male WT and KO islet samples. Differential expression analysis showed that the expression of only 30 genes was significantly affected (p_adj_<0.05), with 9 genes being upregulated and 21 genes downregulated in *Zfp92* KOs (Fig 4A and 4B and S2 Table). Notably, there was a very pronounced increase in expression of *Capn11* gene (Log_2_FoldChange = 4.39), a gene that encodes a calcium-dependent protease calpain 11 that is usually expressed only specifically in testis [33]. *Zfp92* was also increased in KO samples suggesting that ZFP92 may repress its own expression. The downregulated genes included the transcriptional regulators *Mafb*, *Mamld1*, *Cited2*, and *Tref1*, the former of which is known to be enriched in pancreatic α-cells in adult islets where it regulates the expression of pancreatic hormone glucagon (*Gcg*) [34]. To validate RNA-seq findings, we performed RT-qPCR on islet RNA and confirmed that the expression of *Capn11* was increased more than 200 times, and that expression of *Zfp92* is significantly upregulated and *Mafb* is downregulated in samples from KO mice (Fig 4C). While expression of *Gcg* was also decreased, and similar to the RNA-seq result, the changes were not significant. With the exception of *Capn11*, *Mafb*, and a few other genes, these results indicate that *Zfp92* contributes in only a modest way to gene expression in adult islets.

### Blood glucose and *Mafb* expression are decreased in *Zfp*92 KO mice at P1

**Fig 5:**
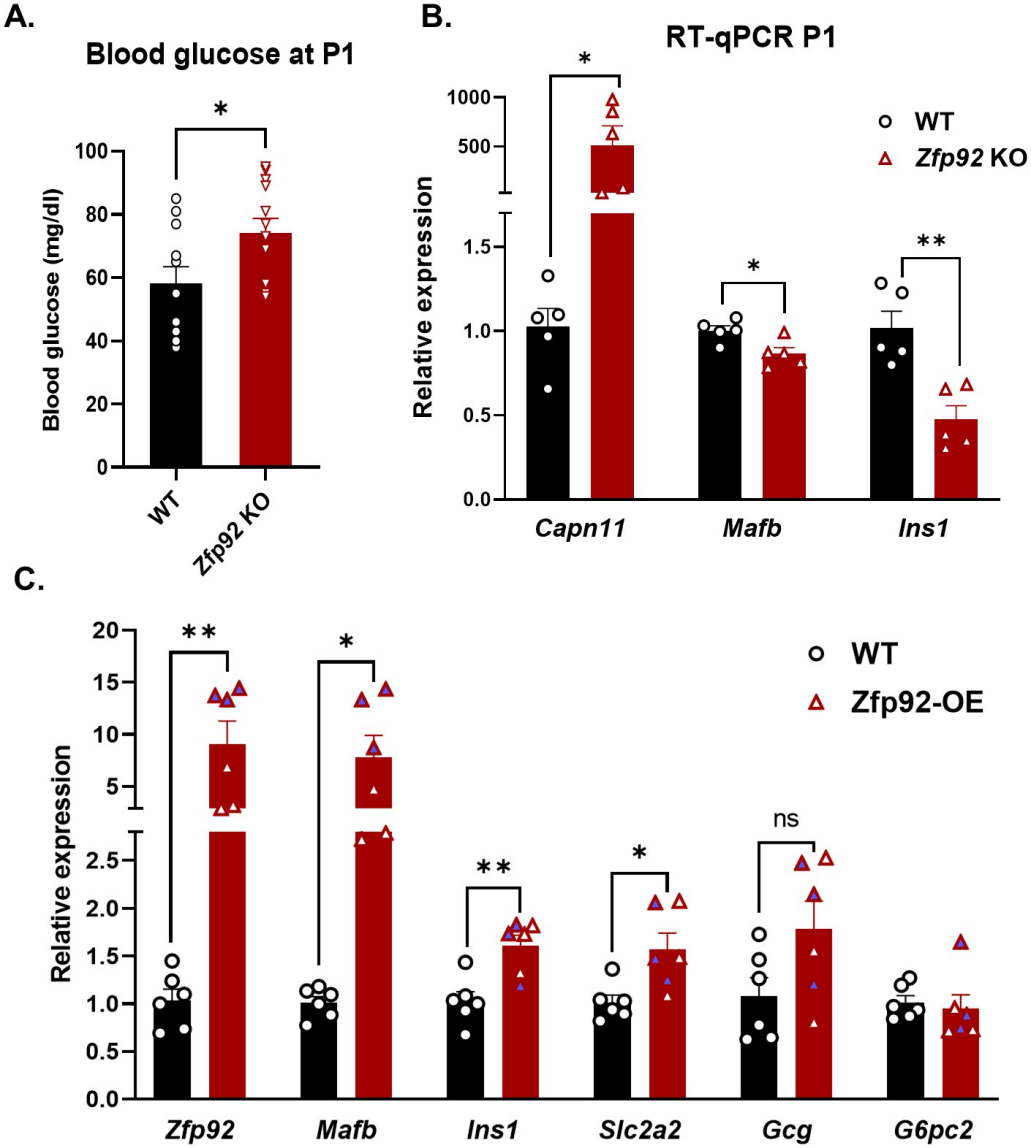
*Zfp92* KO mice have increased blood glucose and decreased *Mafb* and insulin expression at P1. **A**) Random fed blood glucose concentration measurements in wild type (WT) and *Zfp92* knockout (KO) animals show increased blood glucose levels in KO pups at P1 (N = 11,12). **B**) RT-qPCR analysis of mRNA expression for select genes in pancreatic RNA samples collected from newborn pups (P1). *Zfp92* wild type (WT), and knockout (KO) samples (N = 5). **C**) RT-qPCR analysis of mRNA expression for select genes in MIN6 mouse β-cell line that was infected with lentivirus to overexpress *Zfp92* (*Zfp92*-OE) and control cells (WT) (N = 6). The samples where *Zfp92* was increased >10 times are shaded light blue. Error bars: ± SEM. **p≤0.01; *p≤0.05. p-value is determined by an unpaired t-test.

In mice, the transcription factor *Mafb* is critical for the terminal differentiation of β cells during development through regulation of key β-cell genes. After birth, the function of *Mafb* in β-cells is largely replaced by *Mafa*, a closely related transcription factor [35]. Indeed, pancreas-specific *Mafb* KO mice display deficiencies in β-cell development and associated increased blood glucose levels at birth, but these defects are resolved by three weeks of age due to increased *Mafa* expression [36]. To determine whether the absence of *Zfp92* and related decrease in *Mafb* expression affects early endocrine development, we analyzed blood glucose levels and gene expression in the pancreata of postnatal mice at P1 (postnatal day 1) (Fig 5A and 5B). We observed an increase in blood glucose concentration and a decrease in expression of *Mafb* and insulin *(Ins1*) at P1 in KO mouse samples (Fig 5A and 5B). These results indicate that pancreatic β-cell development is compromised in the absence of *Zfp92* due to its effect on *Mafb* expression. As in adult islets, there was also a very strong increase in *Capn11* expression in one day old *Zfp92* KO neonates (Fig 5B). To confirm that ZFP92 regulates *Mafb*, we overexpressed *Zfp92* in MIN6 cells, a mouse β-cell line, and observed a strong increase in *Mafb* expression. In addition, the expression of *Ins1* and *Slc2a2*, two *Mafb* target genes [37], was also increased. These data all point to ZFP92 functioning as a positive regulator of *Mafb* expression.

### De-repression of an IAPez transposable retroelement in intron 3 of *Capn11* in the absence of *Zfp*92

**Fig 6:**
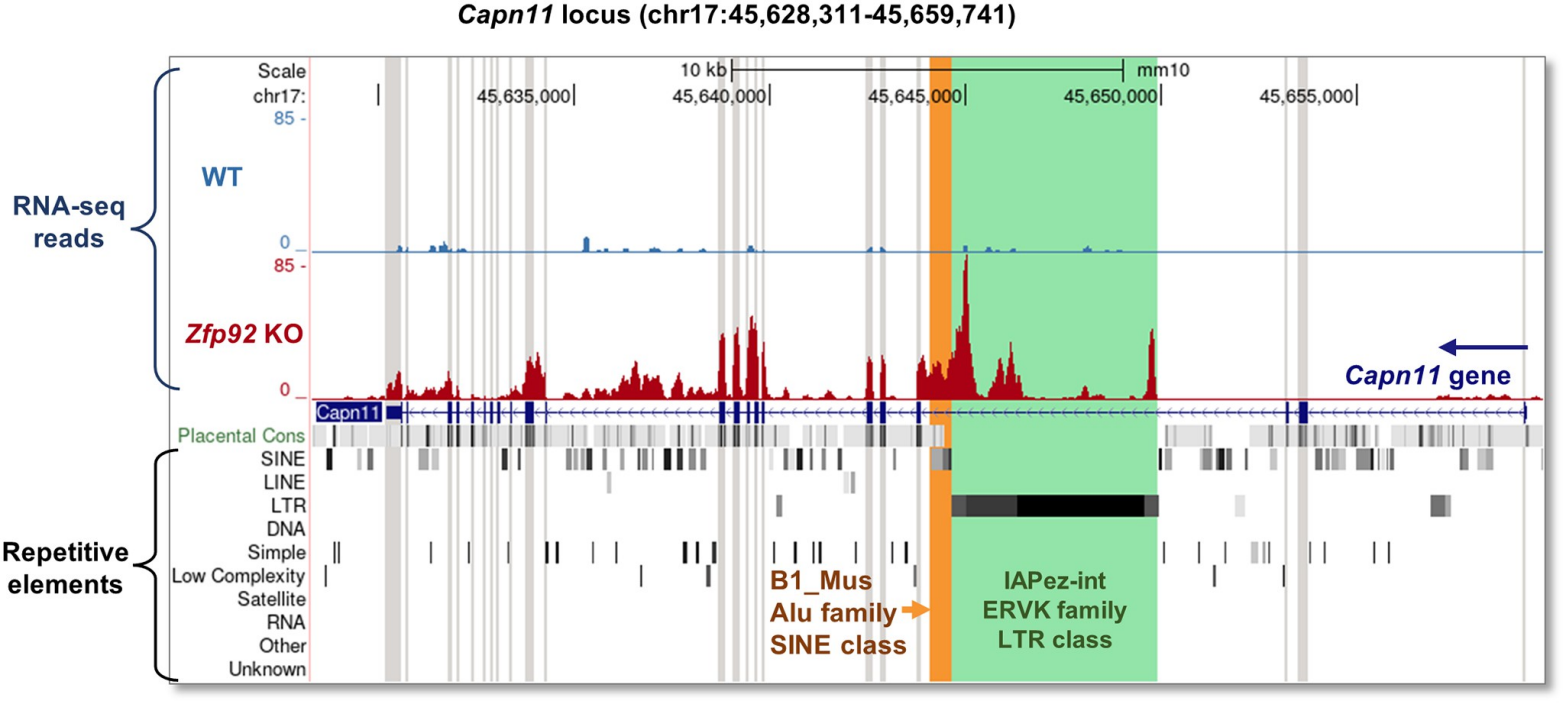
IAPez ERVK LTR retroelement in *Capn11* gene intron 3 is de-repressed in *Zfp92* KO islets. UCSC Genome Browser view of *Capn11* gene region on the mouse chromosome 17 (GRCm38/mm10 assembly). Alignments of RNA-seq sequenced reads for the representative WT (blue) and *Zfp92* KO (red) islet samples are displayed by density graphs and show a strong increase in expressed reads in intron 3 of *Capn11* gene (encoded on the opposite DNA strand) and in further downstream exons of *Capn11*. Full view of RepeatMasker tracks shows that this region is occupied by IAPez ERV consisting of IAPLTR1, IAPez-int repeat elements (ERVK family, LTR class) and adjacent B1_Mus1 Alu-repeat SINE element that appear to be expressed and therefore de-repressed in *Zfp92* KO islets. *Capn11* gene exons are shaded in light gray, SINE element—light orange, IAPez-int element—light green.

The striking increase in *Capn11* gene expression in *Zfp92* KOs prompted us to examine the alignment of RNA-seq reads in relation to the *Capn11* gene. We observed an increase in mRNA reads in *Zfp92* KO samples that maps to a location more than 10 kb downstream from the *Capn11* transcription start site, with exon alignments starting only from exon 4 (Fig 6). Notably, there is an increase in expressed reads present in intron 3 preceding exon 4. Examination of RepeatMasker annotation revealed that these reads align with an IAPez TE consisting of IAPLTR1-Mm1 and IAPez-int repeating elements (ERVK family; LTR class) and adjacent small SINE repetitive elements (B1_mus2 and B1Mus_1, B3A, PB1D10; B1/Alu and B2 families; SINE class). Intracisternal A particles (IAPs) are LTR-containing ERV sequences belonging to an active class of TEs that can compromise genomic integrity and affect genome regulation by providing *cis*-regulatory modules [38]. Considering that many KRAB-ZFPs are linked to repression of TEs, these data suggest that ZFP92 functions to repress TEs within the intron 3 of *Capn11* gene on chromosome 17 in mice. In the absence of *Zfp92*, the IAPez retroelement is re-activated and this, in turn, results in the marked expression of downstream *Capn11* gene sequences. Since our qPCR primers for *Capn11* were designed to amplify exons 4 and 5, we were able to detect a strong increase in *Capn11* expression in *Zfp92* KO mice (Figs 4C and 5B). RT-qPCR with primers designed to amplify exons 1–2 of *Capn11* gene did not yield any product in islet samples, confirming that *Capn11* gene is activated only downstream of IAPez insertion. To investigate whether IAPez-driven *Capn11* expression is de-repressed in other tissues besides pancreatic islets, we performed RT-qPCR on RNA from WT and KO samples from whole pancreas, brain, lung, muscle, intestine testis, and liver (S3 Fig). These results show that while IAPez-driven *Capn11* expression is increased to varying degrees in multiple tissues, the largest increase is in pancreatic islets, indicating that *Zfp92* represses *Capn11* IAPez activity in a cell type-specific manner. Further, to assess changes in global IAPez expression, we designed primers along different regions of IAPez from *Capn11* locus (S4A Fig). RT-qPCR analysis with these primers on islet RNA samples did not reveal any differences in expression between WT and KO samples (S4B Fig), suggesting that ZFP92 represses IAPez in a genomic region-specific manner.

### *Zfp*92 KO-activated IAPez LTR and B1/Alu SINE retroelements form a fusion gene transcript with *Capn11* gene

**Fig 7:**
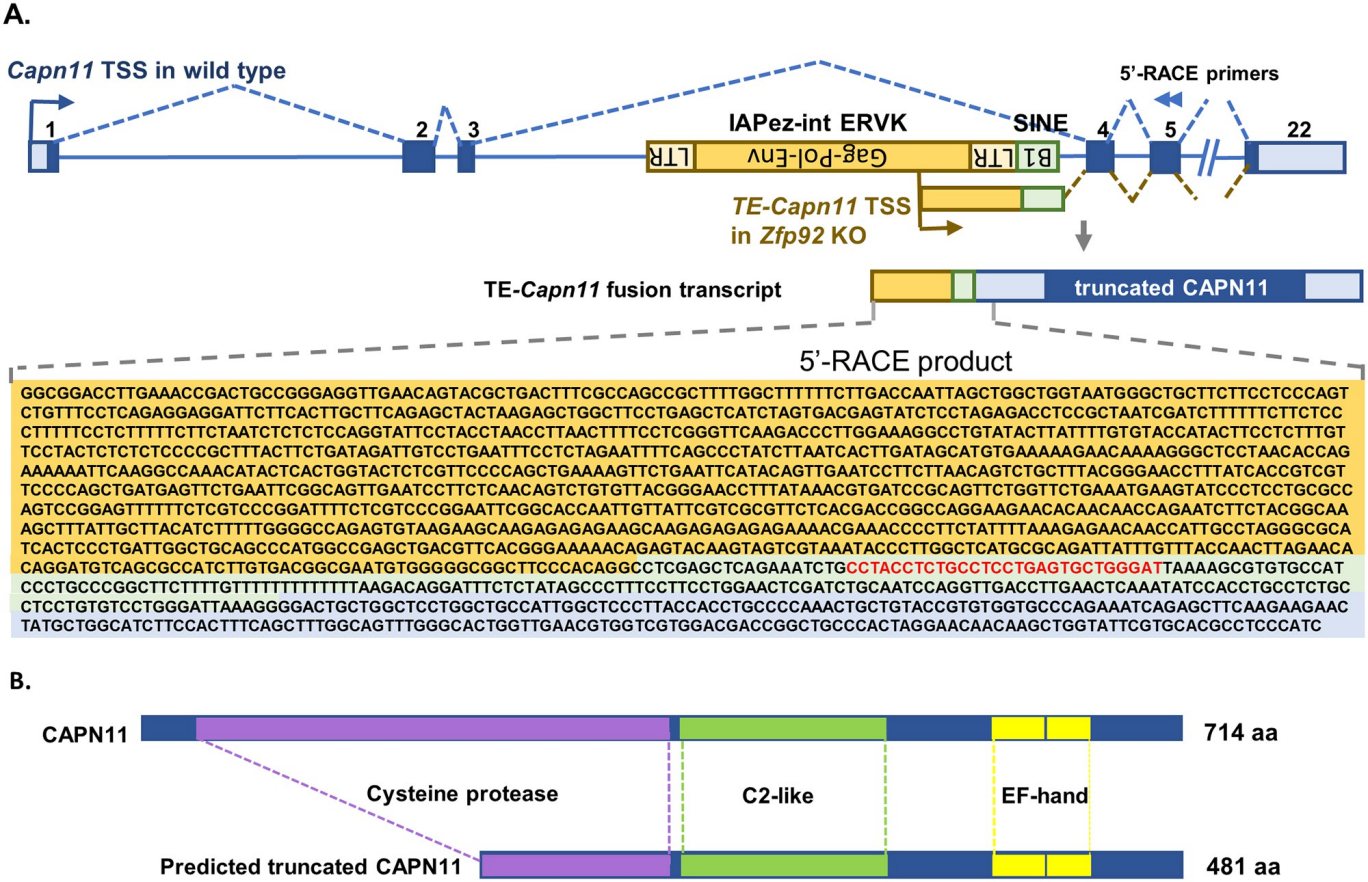
De-repressed IAPez LTR retroelement sequences drive the expression of a fusion TE-*Capn11* transcript in *Zfp92* KOs. **A**) 5’-RACE analysis of mRNA from *Zfp92* KO islet sample revealed the production of a TE-*Capn11* fusion transcript when the IAPez ERVK LTR element is derepressed. The panel shows a schematic representation of *Capn11* gene (blue) and a relative location of B1 SINE (light green) and IAPez ERVK LTR (tan) TEs within the *Capn11* intron 3, location of 5’ RACE primers (blue arrowheads), schematic, and the sequence of obtained 5’RACE product that represents fusion transcript. The IAPez-derived sequence is highlighted in tan, B1 SINE-derived sequence–in light green and a *Capn11* downstream exon sequence—in blue. ZFP92 binding sequence is in red font. **B**) Structure of the full-length CAPN11 and N-terminally truncated CAPN11 protein that is encoded by the open reading frame within the TE-Capn11 fusion transcript.

To further define how re-activation of an IAPez TE in *Zfp92* KO mice drives the expression of *Capn11* mRNA, we performed rapid amplification of 5’ cDNA ends (5’-RACE) on RNA from WT and KO islet samples using reverse primers in exon 5 of *Capn11* gene (Fig 7A). While the WT samples did not yield specific PCR products, the KO sample yielded a 1538 bp product that contained a portion of IAPez and immediately adjacent B1/Alu SINE transposable elements that are then spliced to the exon 4 and 5 of *Capn11* gene (Fig 7A). These data indicate that ZFP92 represses these TEs in *Capn11* locus and, in the absence of this KRAB-ZFP, the IAPez element is activated and acts as a novel promoter region that drives the expression of a fusion TE-*Capn11* transcript. The full-length TE-*Capn11* transcript contains an open reading frame for an N-terminally truncated 481 aa form of CAPN11 protein (Fig 7B), however, we were unable to detect any specific protein bands by Western blots with antibodies raised against human CAPN11 gene (S5 Fig). These data suggest that ZFP92 binds to the TEs in intron 3 of *Capn11* gene, producing a repressive chromatin environment that blocks IAPez expression. In the absence of *Zfp92*, the IAPez is de-repressed and serves as an alternative promoter that drives transcription of a novel IAPez-*Capn11* transcript.

### Dysregulation of LINEs and LTR elements in *Zfp*92 KO islets

**Fig 8:**
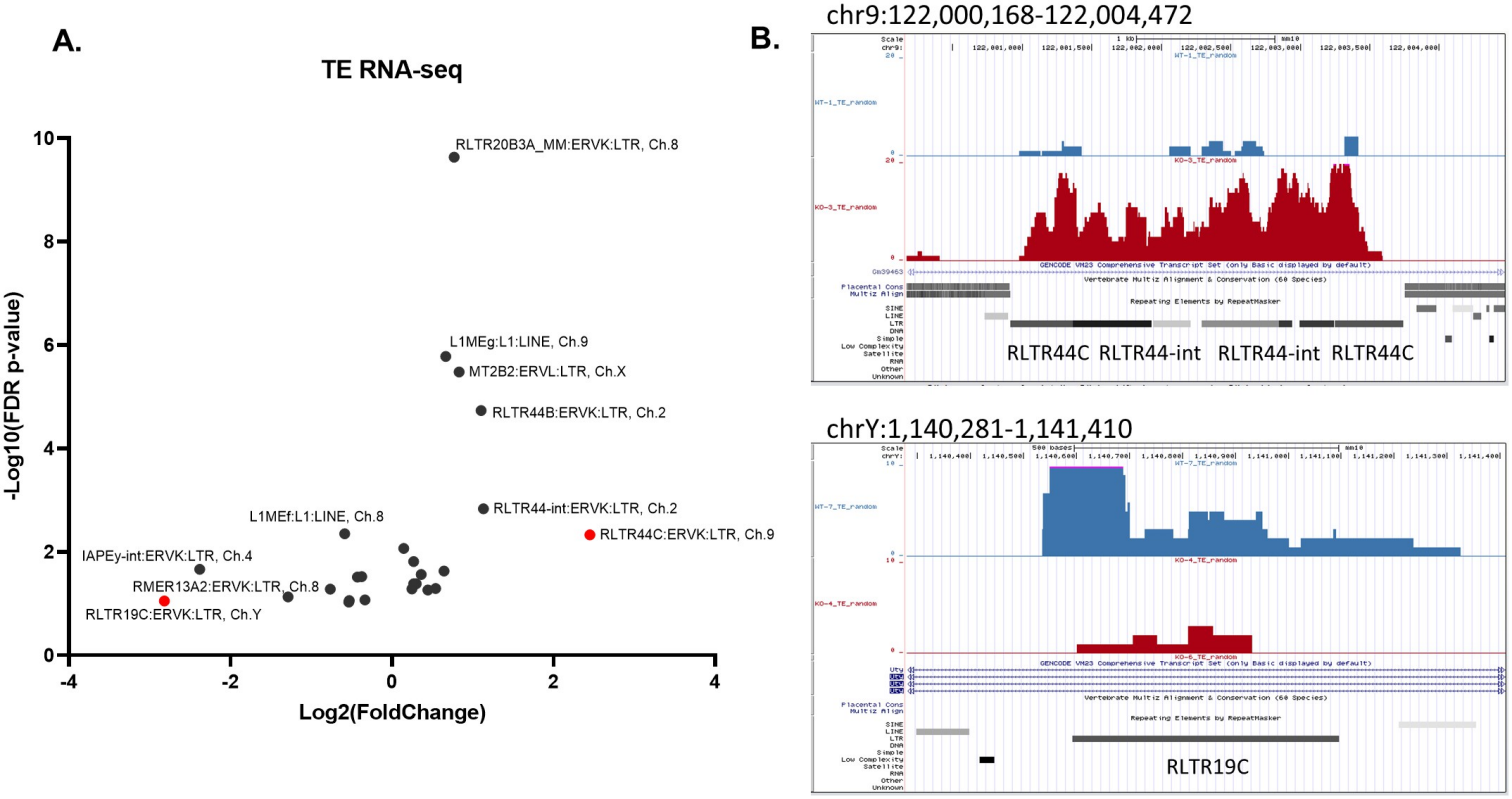
RNA-seq analysis against the TE database shows dysregulation of LINE and LTR retroelements in *Zfp92* KO islets. **A**) Volcano plot showing differentially expressed TEs in *Zfp92* KO vs WT islets collected from 15 week-old male mice (N = 4). RNA-seq read alignment was done by using random assignment of multi-mapped reads to the TE database (RepeatMasker annotations) and differential expression analysis was done for each individual chromosome separately. The volcano plot only shows the TEs whose expression was significantly changed (FDR p_adj_-value <0.1). TEs are labeled by name:family:class and chromosome (Ch) for which differential expression was identified. Top differentially expressed TEs with Log_2_(Fold Change) higher than 2 are labeled in red. **B**) UCSC Genome Browser view of representative top upregulated (RLTR44C) and downregulated (RLTR19B) TEs on the mouse chromosomes 9 and Y, respectively (GRCm38/mm10 assembly). Alignments of RNA-seq sequenced reads for the representative WT (blue) and *Zfp92* KO (red) islet samples are displayed by density graphs.

To determine whether *Zfp92* influences the expression of additional TE elements we re-analyzed islet RNA-seq data by using the TE database and randomly assigning multi-mapped reads. To quantify the findings, we performed differential expression (DE) analysis both on a global and individual chromosome basis. The global DE analysis suggests that expression of 11 TEs is altered (p_adj_<0.1). Among them, RLTR44B showed the largest increase (S6A Fig and S3 Table). However, similar to our qPCR results, there is no change in expression of IAPez TEs at a global level. Considering the very few changes in TE expression and that TE dysregulation might be locus dependent (like the IAPez in *Capn11* locus) we also performed DE analysis at the chromosomal level. These results revealed significant changes in expression of 25 TEs, including ERVs and LINE elements, with the top dysregulated TEs all belonging to ERVK (or ERV2) family of LTR retrotransposons (Fig 8A and S3 Table). Specifically, reads attributable to RLTR44 ERVK-related sequences (RLTR44C, RLTR44-int, RLTR44B) on chromosomes 9 and 2 showed high increases. At the same time, most downregulated TEs were also from the ERVK family including RLTR19C and IAPEy-int elements on chromosomes Y and 4, respectively. Manual inspection of the read alignments for selected individual RLTR44 TEs on chromosome 9 and RLTR19C TE on chromosome Y confirmed respective strong increase or decrease in mapped reads at unique locations (Fig 8B). However, many other retroelements of the same kind in other locations on the same or different chromosomes either did not have any expressed reads or did not show any visible change in expression (S6B Fig). These results indicate that in the absence of *Zfp92*, there is a genome-wide dysregulation of retroelements and that their expression varies both by the specific retroelement and by the genomic context of the retroelement.

### Genome-wide analysis of ZFP92 binding reveals that ZFP92 preferentially binds to B1/Alu SINE elements

**Fig 9:**
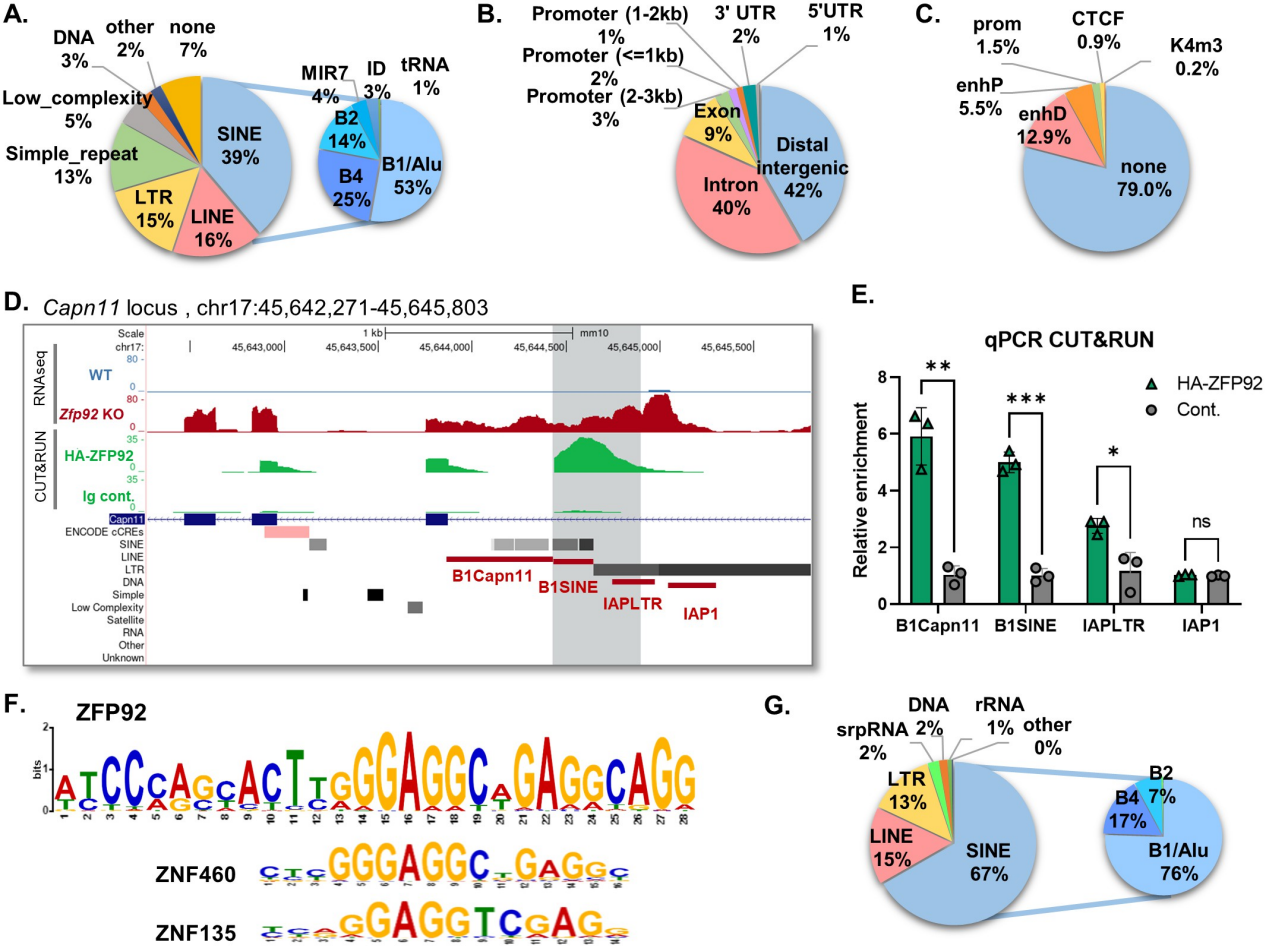
Genome-wide analysis reveals ZFP92 binds preferentially to B1/Alu SINE elements. **A**) Pie chart showing the percentage distribution of different TE classes within the ZFP92 binding peaks obtained by CUT&RUN analysis. 93% of peaks have TEs with SINE elements being the largest group. The inset pie chart shows the family distribution of ZFP92-bound SINEs, with Alu/B1 family being most represented. **B**) Pie chart showing the distribution of ZFP92 binding peaks within different genomic features. **C**) Pie chart showing the distribution of different ENCODE Candidate Cis-Regulatory Elements (cCREs) within the ZFP92 binding peaks. enhD, distal enhancer signature; enhP, proximal enhancer-like signature; prom, promoter-like signature; CTCF, CTCF-only; K4m3, DNase-H3K4me3. **D**) UCSC Genome Browser view of *Capn11* locus showing ZFP92 binding peaks and RNA-seq reads in WT and *Zfp92* KO samples (GRCm38/mm10 assembly). The main peak containing the B1 SINE element is highlighted in gray, with red lines denoting amplicons and primer pairs that were used for qPCR shown in (**E**). **E**) qPCR on CUT&RUN chromatin samples with the primers shown in (**D**) to different regions of Capn11 locus and TE elements found there. (N = 3) Error bars: ± SEM. **p≤0.01; *p≤0.05. p-value is determined by an unpaired t-test. **F**) ZFP92 binding motif predicted by MEME analysis of ZFP92 binding peaks. Under the ZFP92 motif are the two similar motifs (ZNF460 and ZNF135) that were identified by TomTom analysis from the MEME suite. **G**) Pie chart showing the distribution of ZFP92 binding motif within TE elements in ZFP92 peaks. The inset pie chart shows the family distribution of SINEs with FP92 motif, with Alu/B1 family being most represented.

To identify binding sites for ZFP92 we performed CUT&RUN (Cleavage Under Targets & Release Using Nuclease) experiments on MIN6 cells overexpressing HA-tagged ZFP92 protein. Next generation sequencing and data analysis of the resulting chromatin fragments revealed more than 500 ZFP92 binding peaks (q value<0.05). These peaks were further analyzed to determine whether they contain TEs, other genomic features, or ENCODE Candidate Cis-Regulatory Elements (cCREs) [39] (Fig 9A–9C and S4 Table). We found that 93% of the ZFP92-bound peaks contained one or more TEs with SINE elements being the most common (39%), followed by LINE elements (16%), LTR class (15%) of retroelements, and simple repeats (13%). Within the SINE element-bound peaks, the majority (53%) contained B1 type of Alu family of SINEs (Fig 9A). Enrichment analysis of specific TE classes and families in binding sites relative to their genomic distribution showed that SINE elements are the most enriched class (5.2 FoldChange (FC)), followed by DNA elements (2.2 FC) and LTR elements (1.2 FC), while LINE elements show negative enrichment (0.8 FC). Among the families of TEs, the most highly enriched families are B1/Alu (8.2 FC), B4 (4.6 FC) and MIR (3.1 FC) SINE elements (S4 Table). Notably, many of the ZFP92-bound peaks are located in gene-rich regions, with 40% of the peaks located within gene introns, 9% within exons, 6% within extended gene promoters, and 1% within 3’-UTRs (Fig 9B). Moreover, almost 13% of ZFP92-bound peaks contain distal enhancers, 5.5% proximal enhancers and 1% CTCF sites (Fig 9C). Importantly, one of the most enriched ZFP92 binding peaks is in the *Capn11* gene intron 3, with the summit of ZFP92 binding peak being located over the B1/Alu SINE element adjacent to the IAPez element (Fig 9D). The peak extends further into IAPez element that becomes re-activated in the absence of ZFP92. qPCR of chromatin from independent ZFP92 CUT&RUN pulldowns with primer pairs specific to different regions under the ZFP92 peak showed the strongest enrichment for the *Capn11* intron and B1 element, with less enrichment for IAPLTR, and no enrichment for the IAPez body (IAP1), confirming our chromatin sequencing data (Fig 9D and 9E).

We next used the MEME motif-discovery tool to analyze the ZFP92 peaks that revealed a 28 bp consensus ZFP92 binding sequence (Fig 9F). Comparison of the consensus ZFP92 binding motif with those predicted based on the protein sequence of ZFP92 [40] shows only minimal similarities within repeated ‘GAG’ sequences (S7A Fig) highlighting limitations of the latter approach. BLAST alignment search of ZFP92 motif against Dfam TE database [41] retrieved matches to a part of B1 Alu elements, further suggesting that these elements are the main target of ZFP92. Comparison of the ZFP92 binding motif against a database of known motifs revealed similarities to binding motifs for ZNP460 and ZNF135 (Fig 9F), two human KRAB-ZFPs that bind to SINE elements [24]. Moreover, 67% of ZFP92 binding motifs in ZFP92 peaks occur within SINEs, of which 76% are in B1/Alu elements, 17% in B4 elements and 7% in B2 elements (Fig 9G). The ZFP92 binding site is also found in 14% of LTR elements and 12% of LINE elements (Fig 9G and S7B Fig) indicating that SINE-derived sequences are present in some of these elements as well. Inspection of the RNA-seq tracks that span SINE element-specific ZFP92 peaks shows that expected de-repression of SINEs in *Zfp92* KO samples does not lead to an increase in transcription of SINEs. Similarly, neighboring LINE and LTR elements are also often not de-repressed (S8A Fig). However, the absence of *Zfp92* causes some LTR elements to be de-repressed in a context-dependent manner, such as in the *Capn11* locus (Fig 9D) and a few other loci (S8B Fig). These observations confirm our RNA-seq analysis of TE expression highlighting the locus-dependent nature of TE dysregulation in the absence of *Zfp92*.

Combined, our chromatin binding data indicate that ZFP92 binds to multiple TE elements with preferential binding to B1/Alu SINE elements. In the absence of Zfp92, chromatin changes may lead to de-repression of select retroelements in a locus-dependent manner. The ZFP92 binding peaks and corresponding TEs are often found within the gene bodies and may contain regulatory elements, indicating that ZFP92 might function not only as a repressor of TEs but as a potential regulator of gene transcription.

### ZFP92 binds within and affects the expression of Sox17 and Acacb genes

**Fig 10:**
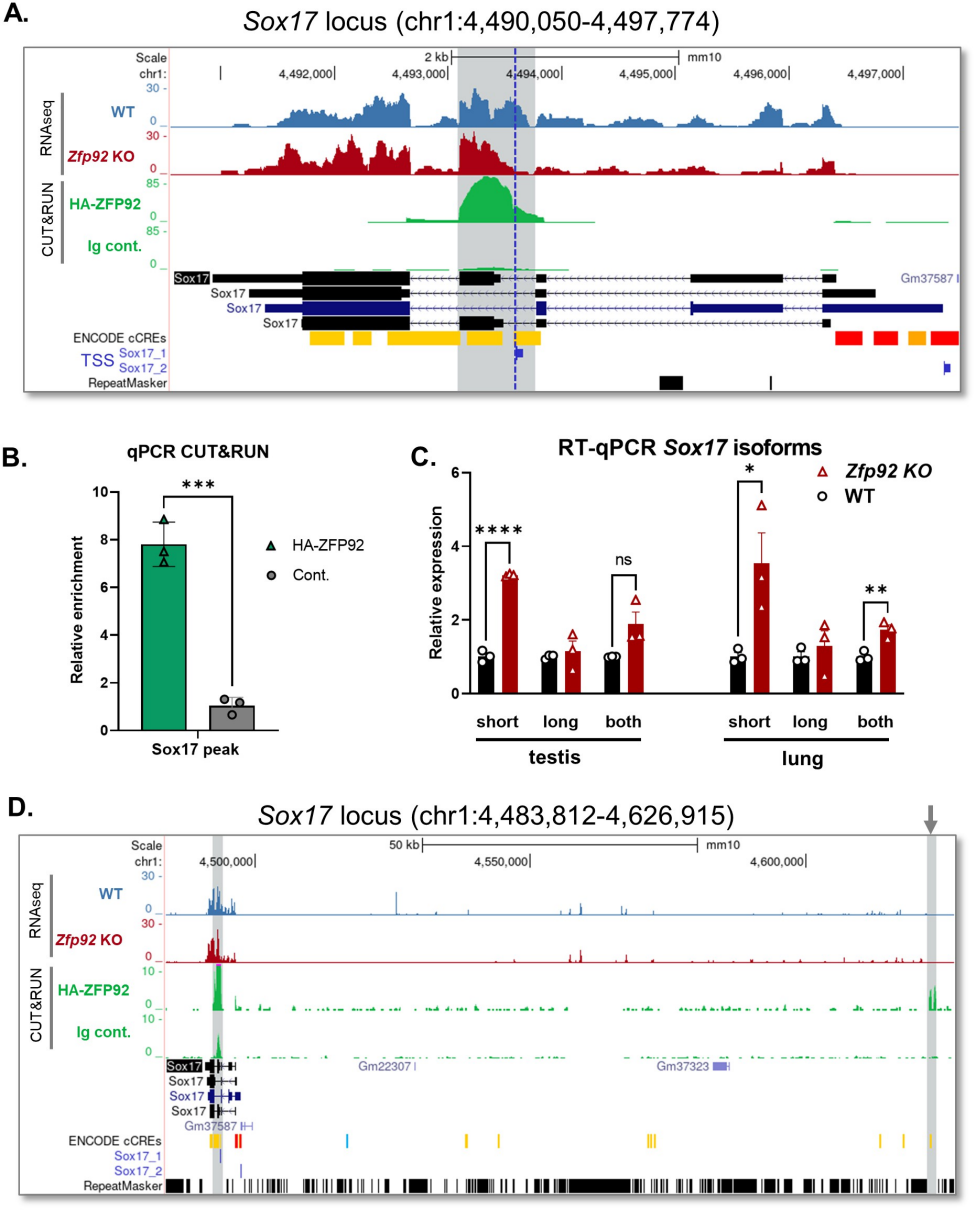
ZFP92 binds within the *Sox17* locus and may regulate expression of the short form of *Sox17* mRNA. **A**) UCSC Genome Browser view of S*ox17* locus showing ZFP92 CUT&RUN binding peak and RNA-seq reads for mRNA expression in WT and *Zfp92* KO samples in *Sox17* locus. The peak is highlighted in gray and covers the extended *Sox17* exon 4, the boundary of which is indicated by the dashed blue line defined by the transcription start site for the short *Sox17* isoform (TSS Sox17-1), and an alternative *Sox17* promoter preceding it. TSS for Sox17 short (Sox17-1) and long (Sox17-2) isoforms are from the Promoters from the EPD track in the UCSC browser (GRCm38/mm10 assembly). **B**) qPCR on CUT&RUN chromatin samples with the primers for the *Sox17* peak (extended *Sox17* exon 4) shows strong enrichment at the locus. (N = 3) Error bars: ± SEM. **p≤0.01; *p≤0.05. p-value is determined by an unpaired t-test. **C**) RT-qPCR analysis of different *Sox17* isoforms expression in testes and lung tissues of WT and *Zfp92* KO mice shows an increased expression of the short *Sox17* isoform. Primers specific to extended exon 4 amplify the short *Sox17* isoform (short), exons 1,2 –long *Sox17* isoform (long), and exons 4,5 –both isoforms (both). (N = 3) Error bars: ± SEM. **p≤0.01; *p≤0.05. p-value is determined by an unpaired t-test. **D**) UCSC Genome Browser view of S*ox17* locus showing two distant ZFP92 binding peaks in *Sox17* locus. The peaks are highlighted in gray, the distal peak is located -128kb upstream of the main peak within extended *Sox17* exon 4 and lies within a conserved DNA sequence that contains a distal enhancer signature (cCRE track) (GRCm38/mm10 assembly).

Strong ZFP92 binding peaks were identified within the *Sox17* and *Acacb* genes (S4 Table). To determine the potential significance of these bindings we further investigated both genes. *Sox17* encodes a transcription factor that is important for early endoderm, vascular and hematopoietic cell development [42]. While *Sox17* is expressed in islets at low levels, RNA-seq did not show any difference in total mRNA expression between WT or KO islets (S2 Table). The ZFP92 binding peak in *Sox17* is located within the exon 4 and an alternative second promoter region that was shown to drive the expression of the short form of *Sox17* mRNA containing only the extended exon 4 and exon 5 [43] (Fig 10A and 10B). To determine whether binding of ZFP92 to this region may affect expression of different *Sox17* mRNA forms, we performed RT-qPCR on mRNA from WT and *Zfp92* KO lung and testes, tissues where *Sox17* is expressed at high levels in adult animals. This experiment revealed an increase in expression of the short *Sox17* form in *Zfp92* KO tissues with no change in expression of the long *Sox17* form that contains exons 1 through 5 (Fig 10C), suggesting that ZFP92 represses expression of the short form of *Sox17*. Although there are no TEs recognized by the Repeat Masker within the *Sox17* ZFP92 binding peak, there is a stretch of sequence (GAGGCAGG) within the extended exon 4 that is part of the identified putative ZFP92 binding sequence. In addition, a second strong ZFP92 binding peak is located 128 kb upstream of the *Sox17* gene (Fig 10D). This second peak is located within conserved sequences that may be involved in formation of regulatory chromatin loop with the ZFP92-bound alternative *Sox17* promoter, further suggesting a yet-to-be-defined regulatory function for ZFP92.

**Fig 11:**
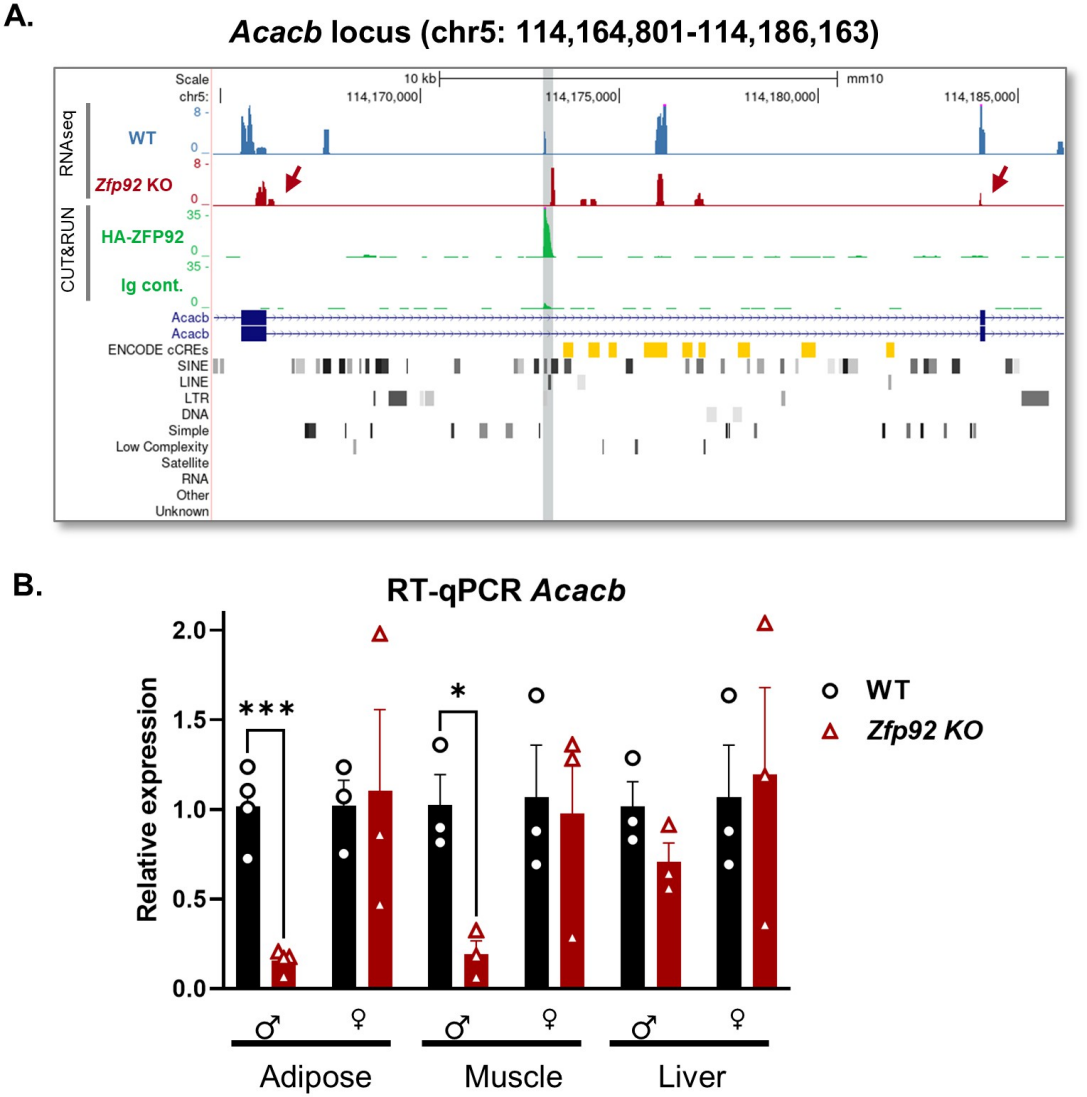
ZFP92 binds a B1/Alu SINE element within the *Acacb* locus and affects the expression of *Acacb* mRNA in adipose and muscle tissues of male mice. **A**) UCSC Genome Browser view of *Acacb* locus showing ZFP92 CUT&RUN binding peak and RNA-seq reads for mRNA expression in WT and *Zfp92* KO samples in *Acacb* locus. The peak is highlighted in gray and contains B1/Alu SINE, as well as LINE and LTR elements (see S7 Fig for a close up). The expression levels of exons surrounding the ZFP92 binding site (arrows) appear to be reduced. **B**) RT-qPCR analysis of *Acacb* mRNA expression in adipose, muscle and liver tissues from male and female WT and *Zfp92* KO mice. Decreased expression of *Acacb* is observed in *Zfp92* KO male adipose and muscle tissues. (N = 3,4) Error bars: ± SEM. **p≤0.01; *p≤0.05. p-value is determined by an unpaired t-test.

Another strong ZFP92 binding peak was identified in an intron of *Acacb* (Fig 11A) that coincides with a PB1D10 B1/Alu SINE element, an overlapping MT2B2 LTR element, and an L1 LINE element (S7A Fig). While we did not observe significant differences in *Acacb* expression in islets (S2 Table), where it expressed at very low levels, there was an observable decrease in RNA-seq aligned reads for exons surrounding the ZFP92-bound region in the *Zfp92* KO sample (Fig 11A). *Acacb* encodes acetyl coenzyme A carboxylase β, the rate-limiting enzyme in fatty acid β-oxidation, and a decrease in *Acacb* expression is associated with increased lipolysis and insulin sensitivity [44]. Since our physiological studies revealed sex-based differences in fat accumulation and insulin sensitivity in mice fed a HFD, we explored whether they could be due to changes in *Acacb* expression by analyzing mRNA from WT and KO male and female mice from three tissues associated with body fat metabolism and insulin sensitivity: white adipose, muscle, and liver. RT-qPCR with primers to the *Acacb* exons surrounding the ZFP92 peak showed a decrease in expression of *Acacb* mRNA in adipose and muscle tissues from male *Zfp92* KO but not in female KO tissues (Fig 11B). These results indicate that in the absence *Zfp92* there is a decrease in expression of *Acacb* in males that may have contributed to the observed increased insulin sensitivity in *Zfp92* KO male mice and higher fat accumulation in KO female mice on a HFD.

## Discussion

In this study, we show that *Zfp92*, a gene encoding KRAB-ZFP that is highly expressed in mouse pancreatic islets, preferentially binds to B1 SINE transposable elements, and is involved in regulation of other nearby retroelements activity and gene expression, including the expression of endocrine cell-defining transcription factor *Mafb* in islets and a regulator of fat metabolism *Acacb* in adipose and muscle tissues.

Murine *Zfp92* is located on the X chromosome and exhibits relatively high sequence conservation among placental mammals where 69 out of 89 (or 77%) of placental mammals have a *Zfp92* ortholog (ensembl.org). Mouse ZFP92 protein is 61.8% identical to its human ortholog that is also located on the X chromosome. Alignment of ten ZFP92 ortholog proteins from species representing different mammalian branches shows that the first 8 zinc fingers are well conserved with the 9th zinc finger showing more variation and being conserved only in rodents (S9 Fig). Our qPCR data and as well as *Tabula Muris* scRNA-seq data indicate that mouse *Zfp92* is highly expressed in pancreatic endocrine islet cells, with lower levels expressed in brain, testis, and intestinal tissues. In contrast, gene expression data for human *ZFP92* from the GTEx portal indicate that its expression is higher in ovary, uterus, brain, lung, and adipose tissues. The different expression patterns suggest that, despite the conservation of amino acid sequence, the mouse and human genes have diverged in terms of their tissue expression patterns. This may reflect divergence in their function, especially with respect to the species-specific silencing of transposable retroelements. Considering that retroelements have species-specific evolutionary origins, the species-restricted evolutionary divergence in KRAB-ZFP-TE regulatory relationships may affect specific gene regulatory networks and their associated phenotypes in a species-specific manner [24,38,45]. For example, it was recently shown that LINE-element suppressing KRAB-ZFP *ZNF528* is expressed in human but not in chimpanzee neural progenitor cells and contributes to human brain function through regulation of mitophagy gene SPATA18 [46]. It is possible, therefore, that ZFP92 has some murine-specific regulatory functions, such as those we identified in pancreatic islets and male adipose tissue.

### ZFP92 predominately binds to SINE elements and regulates TE activity

Our chromatin binding and RNA expression data indicate that the main function of mouse ZFP92 is to regulate the activity of specific TEs consistent with an emerging role for KRAB domain containing ZFPs. Most of the binding sites identified for ZFP92 coincide with TEs, with a large proportion of these being B1/Alu type of SINE elements. Mouse B1 SINE elements are ~140 bp in length and like human Alu elements are derived from the signal recognition particle 7SL and contain a promoter for RNA polymerase III-dependent transcription [47]. SINE elements require LINE-encoded proteins for their mobilization, although the SINE retrotransposition rate is estimated to be higher than that of LINEs with some *de novo* insertions being potentially contributing to disease [48]. There are approximately 500,000 B1/Alu SINEs in the mouse genome that comprise approximately 2.7% of mouse DNA. The majority of B1 SINEs and other TEs are silenced through chromatin repression and DNA methylation by DNA methyltransferases (DNMTs) [49, 50]. However, while KRAB-ZFPs play a vitally important role in repressing TEs, few have been identified that bind to SINE elements. Consistent with ZFP92 preferentially binding to B1/Alu SINE elements, the predicted ZFP92 binding motif is a 28 bp sequence that is highly similar to B1/Alu SINEs. While shorter in length, the predicted binding motifs for ZNF460 and ZNF135, two other KRAB-ZFPs that bind SINEs [24], are very similar to that for ZFP92. Our analysis also revealed that while ZFP92 binds predominately in SINE elements, it sometimes also binds to LINE and LTR TEs, indicating that SINE sequence fragments are present in other TEs and, possibly, other genomic features [51]. Recent studies indicate that SINE sequences within the genome can play important functions such as act as enhancers, promote chromatin compartmentalization, and regulate chromatin accessibility through SINE-KRAB-ZFP interactions [52–54].

Interestingly, we did not observe a global increase in SINE expression in *Zfp92* KO tissues, suggesting that the absence of ZFP92 alone is not enough to activate the transcription of silenced SINEs. While it is possible that the methods used to construct the library for RNAseq did not capture small SINE transcripts, we did observe the altered expression of several LINE, LTR elements, and genes in the *Zfp92* KO mice, indicating significant genomic dysregulation in the absence of ZFP92. The most striking example of such dysregulation is the very strong activation of a novel TE-*Capn11* fusion transcript in *Zfp92* KO islets due to the activation of an IAPez retroelement located next to the ZFP92-bound B1 SINE element in the 3^rd^ intron of the *Capn11* gene. In the absence of *Zfp92*, the IAPez ERV in this location is activated and serves as an alternative promoter that drives the robust expression of the TE-*Capn11* transcript that contains IAPez and SINE sequences that are spliced to the downstream *Capn11* exons. This surprising finding provides a very stark example of how de-repression of TEs can lead to creation and strong expression of a brand-new transcript and, potentially, impact cell function. It also indicates that ZFP92 is indeed involved in repressive complexes and its removal leads to chromatin opening and activation of nearby regulatory elements. Interestingly, similar activation of IAPez expression within *Capn11* locus was observed in DNA methyltransferase *Dnmt1* KO mice [55], further suggesting that ZFP92 is a part of TE repressive complex.

Although the novel TE-*Capn11* fusion transcript contains an open reading frame that encodes an N-terminally truncated form of calpain 11, a calcium-dependent protease normally expressed only in adult testis in mice [56], we did not detect any protein product in islets using available antibodies. Despite this, we cannot rule out that the protein can potentially be made in islets or other tissues. While the predicted protein would lack only part of the N-terminal protease domain, so might not be catalytically active, it would still be expected to contain five C-terminal calcium-binding EF-hand domains (Fig 7B). EF-hand domain-containing proteins often act as calcium buffers which control the level of free Ca^2+^ ions in the cytoplasm [57]. It is possible, therefore, that the TE-*Capn11* -transcript-derived CASPN11 protein, even if truncated, could influence function of different tissues. In *Zfp92* KO mice, besides being overexpressed in islets, *Capn11* fusion transcript is highly expressed in brain, intestine, testis, and liver, suggesting that *Zfp92* may regulate the *Capn11* IAPez in a tissue-specific manner. Even though the level of *Zfp92* expression was low in some of these adult tissues, it may have been expressed in progenitor tissues at some point during development. In the absence of *Zfp92*, changes in the chromatin environment and de-repression of IAPez during development could lead to continuous overexpression of TE-*Capn11* transcript at later stages in these tissues.

Global analysis of TE expression in *Zfp92* KOs showed varied increases and decreases in several types of LINE and LTR elements. We also observed that the same types of elements were dysregulated in one position and unchanged in others. Similarly, while some changed elements were in proximity to ZFP92 binding sites, others were not. This suggests that regulation of TEs by ZFP92 is influenced by genomic context, the state of chromatin in the locus, distal chromatin interactions and by chromatin modifying complexes that may interact with this KRAB-ZFP. Indeed, a recent study reports that chromatin modifications affect TE expression in a complex way where not only repressive marks, but widespread marking of TEs by bivalent or activating marks, were shown to influence TE expression and chromatin accessibility. Furthermore, the effects of chromatin modifications are sequence- and context-specific, with different chromatin modifiers regulating the expression and chromatin accessibility of specific subsets of TEs [58–60]. For example, different LTR retrotransposons can also be modulated by different chromatin modifying complexes. RLTR44 is upregulated in the absence of *Zfp92* and is repressed by the polycomb complex [61] suggesting that ZFP92 associates with polycomb or other repressive complexes to repress RTLR44. On the other hand, several IAPey retroelements are downregulated in the absence of *Zfp92*. IAPeys are enriched on the Y chromosome and are active in male germline [62,63]. While KRAB-ZFPs are generally considered to be transcriptional repressors, some KRAB-ZFPs activate gene transcription through interaction with activating complexes [64,65]. In any case, our studies of *Zfp92* add to the growing evidence that IAPs and other LTR transposons act as *cis*-modifiers of gene expression and that KRAB-ZFPs regulate them in locus-, tissue-, species-, and strain-specific manners [25,66–69].

### Role of Zfp92 in islets

The expression of mouse *Zfp92* increases during development as endocrine progenitor cells are converted into nascent β-cells, and then is maintained in adult β-cells. *Zfp92* KO mice fed a regular diet do not display major abnormalities in pancreatic islet structure, insulin secretion, or β-cell effects on glucose homeostasis. However, we did observe a decrease in fasting blood glucose levels in *Zfp92* KO males which may be due to reduced levels of glucagon associated with a decrease expression of *Mafb*, a transcription factor that is important for islet maturation and function. Indeed, among the very few changes observed in the transcriptome of adult *Zfp92* KO islets, there was a verified decrease in *Mafb* gene expression. The absence of an overt β-cell phenotype in adult *Zfp92* KO mice is similar to pancreas-specific *Mafb* KO mice where only a decrease of glucagon secretion is observed in adult mice [36]. *Mafb* is highly expressed in progenitor α- and β-cells, is required for β-cell development, becomes largely replaced by *Mafa* in adult mouse β-cells, and continues to be expressed and regulate glucagon expression in adult α-cells [34,37,70]. Consistent with the role of *Mafb* during β-cell development, we showed that in the absence of *Zfp92*, newborn mice have decreased expression of *Mafb* and *Ins1* genes that is accompanied by increased blood glucose levels. Additionally, *Zfp92* KO mice exhibit increased fasting blood glucose levels when fed a HFD for 12 weeks, indicating a modest impairment of β-cell function under metabolic stress. Together, these results indicate that *Zfp92* contributes to *Mafb* regulation, both during β-cell development and in adult islets.

The mechanism whereby ZFP92 protein promotes the expression of *Mafb* is not clear. We observed only weak ZFP92 binding peaks around *Mafb* promoter although there are several sites of stronger ZFP92 binding within the 20 kb of DNA surrounding *Mafb* gene that either bind to a TE element or to a partially conserved binding motif sequence (S10 Fig). It is also possible that there is a de-regulation of TEs that are more distal to *Mafb* gene that are important for the regulation of *Mafb* in islets. Indeed, several studies have shown that TEs can serve as enhancers, are associated with open chromatin regions, contain transcription factor binding sites, and are involved in cell-type specific chromatin domain organization and gene regulation [71–73]. Alternately, the dysregulation of other genes in endocrine cells that are important for *Mafb* expression could be affected.

### Role of *Zfp*92 in other tissues

While our study began with a focus on pancreatic islets, our physiological studies also revealed a role for *Zfp92* in adipose tissue of male mice. Male KO mice on a normal diet grew at a slower rate and exhibited increased insulin sensitivity and body fat content when on a HFD compared to female KO mice. An increase in total body fat is one of the two significant phenotypes reported by the International Mouse Phenotyping Consortium (IMPC) for an independently derived *Zfp92* KO allele on different mouse strain (C57Bl/6NJ) [74]. The second significant phenotype described for IMPC *Zfp92* KO mice was a decreased bone mineral content, something we did not study.

The sex-specific differences in body fat accumulation and insulin sensitivity in *Zfp92* KO mice on a HFD are likely due to sex-specific changes in *Acacb* gene expression. ZFP92 binds to an array of TE elements that includes B1/Alu SINE element within the intron of *Acacb* gene and deletion of *Zfp92* leads to a male-specific reduction in the expression of *Acacb* in adipose and muscle tissue. By catalyzing synthesis of malonyl-CoA, an inhibitor of rate-limiting step in fatty acid uptake and oxidation, ACACB is thought to be a key regulator of fatty acid oxidation [75,76]. Global deletion of *Acacb* in mice leads to decreased fat accumulation and increased insulin sensitivity due to increased fat metabolism in muscle and adipose tissues [77, 78]. The *Zfp92*-dependent reduction of *Acacb* only in males in our study may explain the greater fat accumulation in KO females and increased insulin sensitivity in male KO mice fed a HFD. The mechanisms responsible for the sex-specific regulation of *Acacb* are not clear, although *Acacb* was previously shown to be more highly expressed in female liver [79] and hypothalamus [80]. Interestingly, in humans a strong QTL influencing increased percent body fat specifically in women is located on chromosome 12q (12q24.3–12q24.32) near *ACACB* [81], further suggesting sex-specific differences in the expression or function of this gene.

In this study, we also showed that ZFP92 is involved in regulation of expression of essential transcription factor *Sox17* [82]. We have previously shown that during early development *Sox17* is expressed in two distinct progenitor cell populations, endodermal and hemogenic endothelial cells, that preferentially express short or long *Sox17* RNA forms driven by two different promoters [43,44]. We discovered that ZFP92 binds to the promoter region for the short RNA form that, in the absence of *Zfp92*, is upregulated in adult lung and testis tissues. We also identified a distal site upstream of *Sox17* that is bound by ZFP92. Additional studies are necessary to determine whether either of these two binding sites has effects on the development of endodermal, vascular, or other lineages that express *Sox17*.

Finally, while we only studied gene and TE expression differences for selected genes and tissues, it is possible that genome-wide chromatin dysregulation due to the lack of *Zfp92* may impact other genes, tissues, and developmental processes. However, the observed mild phenotype of *Zfp92* KO mice indicates that there’s a redundancy among KRAB-ZFPs targeting different TEs. Indeed, mice in which an entire cluster of KRAB-ZFPs is deleted are viable and relatively normal [23].

## Conclusion

Our results indicate that *Zfp92* has diverse functions in the mouse. While it mainly functions to suppress retrotransposons by binding preferentially to B1/Alu SINE elements, it also regulates several genes in pancreatic islets, white adipose and muscle. Furthermore, while mice lacking *Zfp92* are viable, they exhibit sex-specific alterations in insulin sensitivity and fat content. Thus, ZFP92 not only represses TEs, it also regulates the transcription of specific genes in discrete tissues.

## Methods

### Ethics statement

Animal procedures were conducted in accordance with the ethical guidelines of the National Institute of Health (NIH) and approved by the Vanderbilt Institutional Animal Care and Use Committee. This study did not include research with human subjects.

### Mouse lines and husbandry

*Zfp92* KO mice were derived using CRISPR/Cas as previously described [31]. Briefly, guide RNAs (gRNAs) were microinjected into Cas9-expressing embryos heterozygous B6J/129(Cg)-*Gt(ROSA)26Sor*^*tm1*.1(CAG-cas9^*^, *-EGFP)Fezh/J*^ mice (JAX 026179) by the Vanderbilt Genome Editing Resource. Founder animals were then backcrossed to C57Bl/6J mice for 7 generations, segregating the Cas9-expressing ROSA26 allele in the process. Pups were genotyped by PCR using primers surrounding the deletion site (S1 Table).

### Mouse body weight and composition

Male and female mice were weighed weekly between 4 to 14 weeks of age. Mouse body composition was measured at 14–15 weeks by using a nuclear magnetic resonance spectroscopy imaging machine (Bruker Instruments, The Woodlands, TX) at Vanderbilt Mouse Metabolic Phenotyping Center.

### Glucose homeostasis

Blood glucose concentrations were determined in tail blood samples by using a BD Logic glucometer. Intraperitoneal glucose tolerance tests (GTT) were performed following a 16-hour overnight fast. Mice were administered D-glucose (2 mg/g body mass) by intraperitoneal injection and blood glucose concentrations were measured at 0, 15, 30, 60, and 120 minutes. Insulin tolerance testing (ITT) was performed following a 4-hour morning fast by giving 0.1 U/mL of insulin (Humulin R (Eli Lilly) in DPBS (Thermo Fisher)) by intraperitoneal injection and blood glucose concentrations were measured at 0, 15, 30, 60, and 120 minutes. Plasma insulin concentrations were determined in triplicate by radioimmunoassay (Millipore, PI-13K) by the Vanderbilt Hormone Assay and Analytical Services Core.

### Islet isolations

Mouse islets were isolated following an injection of 0.6 mg/mL Collagenase P (Roche) into the pancreatic bile duct. Partially dissociated tissue was fractionated using a Histopaque-1077 (Sigma) gradient followed by hand-picking of islets. Islets were isolated by injection of 0.6 mg/mL Collagenase P (Roche) into the pancreatic bile duct, and partially dissociated tissue was fractionated using a Histopaque-1077 (Sigma) gradient followed by hand-picking of islets. Islet isolations were performed by the Vanderbilt Islet Procurement and Analysis Core.

### RT-qPCR

RNA was isolated from tissues and whole islets from 14–15-week-old mice, from pancreata removed from one day old animals (P1), and from MIN6 cells using Maxwell 16 LEV SimplyRNA Tissue Kits (Promega, TM351). After extraction, RNasin (40 U/uL, Promega) was added to the RNA samples before storage at -80°C. Reverse transcription was done using a High Capacity cDNA Reverse Transcription Kit (Thermo Fisher). 2 ng of cDNA were used in real-time qPCR with Power SYBR Green PCR master mix (Thermo Fisher) using CFX96 Real-Time PCR system (Bio-Rad). Relative expression was determined by the ΔCt method by normalizing to the expression of *Actb* or *Hprt*. Primers are listed in S1 Table.

### RNA-seq

The quality of RNA samples was assessed using an Agilent 2100 Bioanalyzer. Only those samples with an RNA integrity number (RIN) 7 or above were used for sequencing. cDNA libraries were constructed using the Low Input Library Prep Kit pipeline. An Illumina NovaSeq6000 instrument at Novogene Corp was used to produce paired-end, 150 nucleotide reads for each RNA sample. Paired-end sequencing produced approximately 55 million reads per sample, which were processed utilizing Trim Galore 0.5.0 (which relies on Cutadapt 1.18) to remove adapter sequences and pairs that were either shorter than 20 bp or that had Phred scores less than 20. The Spliced Transcripts Alignment to a Reference (STAR v2.6.0c) application [83] was used to perform sequence alignments to the mm10 (GRCm38) mouse genome reference and GENCODE comprehensive gene annotations (Release M17). STAR’s two-pass mapping approach was used to increase the detection of reads mapping to novel junctions identified during the first mapping pass. Overall, approximately 85–87% of the raw sequencing reads were uniquely mapped to genomic sites. For additional sample-level quality control analysis and downstream pairwise comparisons, DESeq2 was employed [84]. For the analysis of TEs, the parameters for STAR were modified to account for the more complex TE content in mice by allowing a higher number of multi-mapped reads [85] (S1 Method). The alignments were then analyzed using the count feature of TE transcripts [86]. In addition, TE annotations were extracted from the UCSC Genome Browser’s RepeatMasker-generated RMSK track [87] which enabled the screening of DNA sequences enriched in interspersed repeats and low complexity DNA sequences. While alignments were run on the entire genome, the TE mapping and read counting were performed both on the whole genome and on a per-chromosome basis. DESEq2 was used for downstream pairwise comparisons. All data processing was performed at the Advanced Computing Center for Research and Education (ACCRE) at Vanderbilt University.

### Zfp92 overexpression studies

MIN6 mouse insulinoma cells (C0018008, AddexBio) were cultured in advanced DMEM medium (C0003-04, AddexBio) supplemented with 15% FBS (Atlanta Biologicals), 1% penicillin /streptomycin and 5 μM of β-mercaptoethanol (both Thermo Fisher) at 37°C, with 5% CO_2_ and 95% humidity. Lentiviral vectors submitted by Didier Trono’s lab were obtained from Addgene (Addgene # 12257, #12260, #12259). To overexpress *Zfp92*, a full-size mouse *Zfp92* cDNA containing N-terminal HA-tag was generated by RT-PCR, cloned into the lentivirus expression vector, then co-transfected with packaging vectors into HEK293T cells using PolyFect reagent (Qiagen). Viral supernatants were harvested 24-48h post-transfection, filtered through a 0.45μm filter, and titered using Lenti-X qRT-PCR Titration Kit (Takara). For the experiment, MIN-6 cells were seeded in 24-well plates at 100,000 cells per well and infected with lentivirus at MOI ~2 then after 72 hours cells were lyzed and RNA was isolated. A similar lentivirus overexpression protocol was used for CUT&RUN experiments.

### Identification of ZFP92 binding sites

HA-ZFP92 lentivirus-infected MIN6 cells were trypsinized and washed 2 times with 1%BSA in PBS solution (150 x 10^3^ cells were used per pull-down). The CUT&RUN (Cleavage Under Targets and Release Using Nuclease) assay was performed by using the CUT&RUN Assay KIT (#86652 Cell Signaling Technology) following the manufacturer’s protocol. For anti-HA-ZFP92 pulldowns from MIN6 cells, rabbit anti-HA antibodies (#3724) and normal rabbit control IgGs from the kit were used (all antibodies are from Cell Signaling). Briefly, buffer-washed cells were bound to Concanavalin A-coated magnetic beads, permeabilized with 0.05% digitonin buffer, incubated with antibodies at 4°C overnight, washed twice with digitonin buffer, and incubated with Protein A-MNase (pA-MN) for 1 hour at 4°C. After two washes, cell-bound beads were incubated for 30 min at 4°C in 2 mM CaCl_2_ for pA-MN digestion, then incubated with RNAse-containing Stop Buffer (30 min. at 37°C) to release DNA. DNA was further purified using spin columns (14209S, Cell Signaling) and directly used for library prep or qPCR. For sequencing, DNA was processed through the ChIP-seq library prep and Illumina NovaSeq6000 PE150 next-generation sequencing pipeline at Novogene Corporation. The sequencing reads were processed with nf-core/cutandrun version 3.0 [88,89], which is an analysis pipeline for CUT&RUN experiments built using Nextflow for portability and reproducibility. FastQC was used for sequencing quality control reporting and Trim Galore for adapter trimming and to remove low quality reads. Bowtie2 [90] was used for DNA alignment against the UCSC mm10 mouse genome reference and gene annotations. Samtools [91] was then used to filter on quality, sort, and index alignments, followed by duplicate removal by Picard [92]. Peak calling was performed with MACS2 [93]. To quantify the frequency of classes and families of repeat elements across the genome, we calculated the total genomic length of each element from the coordinate data available from the RMSK track [87] and aggregated these either by class or family, then divided by the genome length to obtain a coverage ratio. Then, to determine enrichment of such elements within the CUT&RUN peaks, we matched repeat elements to the coordinate regions of the peaks and then aggregated these to determine enrichment frequency. The MEME,TomTom and FIMO tools within the MEME suit were used for the *de novo* prediction of the ZFP92 binding motif within ZFP92-bound peaks, comparison of the ZFP92 motif against a database of known motifs and finding occurrences of the ZFP92 binding motif within the peak TEs, respectively [94].

### Immunofluorescent staining

Whole pancreata were fixed for 4 hours in 4% paraformaldehyde, incubated overnight at 4°C in 30% sucrose, embedded in OCT compound (Tissue Tek), frozen on dry ice, and sectioned at a depth of 8 μm. The staining was performed following standard procedure as previously described [95]. Two primary antibodies were used, a guinea pig anti-insulin (ThermoFisher, 1:1000) and a rabbit anti-glucagon (Linco, 1:1000). Two secondary antibodies used were a donkey anti-rabbit IgG Alexa-488 conjugated and donkey anti-guinea pig IgG Alexa-555 conjugated (ThermoFisher,1:1000). After antibody staining, slides were mounted with Invitrogen ProLong Gold antifade reagent with DAPI (ThermoFisher). Images were acquired using Aperio ScanScope CS imaging system. Quantifications of cell area were done on evenly spaced pancreatic sections at 150 μm apart. Relative β-cell (insulin^+^) and α-cell (glucagon^+^) areas were calculated by dividing the area of hormone^+^ cells by that of the whole pancreas (DAPI^+^). The images shown are representative of the phenotype observed in at least three different animals per genotype.

### Western blot analysis

Tissues were lysed in RIPA buffer (10 mM Tris-HCl, pH 7.5; 140 mM NaCl; 1 mM EDTA; 1% Nonidet P-40; 0.1% sodium deoxycholate; 0.1% SDS) containing protease inhibitors (300 μg/ml phenylmethylsulfonyl fluoride [PMSF], 1X protease inhibitor cocktail (Sigma)). Samples (20 μg of total protein) in 1X Laemmli sample buffer were resolved on a gradient 12% SDS-PAGE and transferred to polyvinylidene difluoride (PVDF) membranes (Millipore). Membranes were blocked with PBST (PBS; 0.02% Tween 20, pH 7.5) containing 5% non-fat dry milk and incubated with primary antibodies overnight. Primary antibodies used were mouse anti-CAPN11 (1:200; MA5-21265, ThermoFisher), and rabbit anti-GAPDH (1:1000; Cell Signaling). After washing with PBST, the membranes were then incubated with a secondary anti-mouse or anti-rabbit HRP-conjugated antibodies (Cell Signaling, 1:2000), and detected with SuperSignal West Dura Extended Duration Substrate chemiluminescence kit (ThermoFisher).

## Supporting information

S1 Fig Analysis of alternate *Zfp92* mRNAs in mouse islets. **A**) Schematic representation of predicted mRNA isoforms 1 and 2 for *Zfp92* gene and relative location of primers used for RT-qPCR assays. **B**) Table listing RT-qPCR assays, primers and expected PCR product sizes for each mRNA form (qPCR cycling protocol). The 4C assay detects both RNA forms. **C**) Image of the gel showing PCR products for each assay after 40 cycles of qPCR. **D**) Quantification of relative expression of *Zfp92* RNA forms 1 and 2 by RT*-*qPCR. The expression of individual mRNA forms was normalized to the common 4C assay. *Zfp92* mRNA form 1 is predominantly expressed in islets. N = 3, error bars: ± SEM. ***p≤0.001. p-value determined by unpaired t-test.
(TIF)

S2 Fig Glucose and insulin tolerance tests in *Zfp92* KO mice. Intraperitoneal glucose tolerance test (GTT) results (**A**) and insulin tolerance test (ITT) results (**B**) comparing regular chow-fed wild type (WT) and *Zfp92* knockout (KO) male and female mice at 14–15 weeks. No differences in GTT or ITT were observed. N = 7–9 for each sex and genotype. **C**) GTT results comparing wild type (WT) and *Zfp92* knockout (KO) male and female mice at 14–15 weeks after 10 weeks on a high fat diet. N = 8 for each sex and genotype (*p≤0.05: WT vs KO males). **D**) ITT results comparing wild type (WT) and *Zfp92* knockout (KO) male and female mice at 14–15 weeks after 10 weeks on a high fat diet. n = 8 for each sex and genotype (**p≤0.01: WT vs KO males). KO male mice have higher insulin sensitivity than WT mice after 10 weeks on HFD. n = 8 for each sex and genotype. (** p≤0.01; *p≤0.05: WT vs KO males). Error bars: ± SEM. **p≤0.01; *p≤0.05. p-value determined by ANOVA.
(TIF)

S3 Fig Expression of re-activated *Capn11* transcript in different tissues of *Zfp92* KO mice. RT-qPCR analysis of expression of IAPez-driven *Capn11* transcript (exons 4–5) in *Zfp92* KO relative to wild type (WT) mouse tissues. N = 4, error bars: ± SEM. ***p≤0.001; *p≤0.05. p-value is determined by an unpaired t-test.
(TIF)

S4 Fig Expression of IAPez ERV sequences in *Zfp92* KO islets. **A**) Schematic representation of IAPez ERV found in exon 3 of *Capn11* gene and relative location of primers used for RT-qPCR assays. LTRs are shown in light gray color, putative ZFP92 binding site is in red. **B**) RT-qPCR analysis of expression of IAPez with the probes shown in (**A**) in *Zfp92* KO relative to wild type (WT) mouse islets. N = 4, error bars: ± SEM. ***p≤0.001; *p≤0.05. p-value is determined by an unpaired t-test.
(TIF)

S5 Fig Western blot analysis of CAPN11 protein *Zfp92* KO islets. Islet lysates from individual *Zfp92* KO and wild type mice were used in Western blot analysis using antibodies raised against the C-terminus of human CAPN11 protein. The testis protein lysate is loaded as a positive control since *Capn11* is highly expressed in testis. The predicted full CAPN11 protein size is ~80kD, however, the protein of this is not detected in the testis sample. No specific protein bands are detected in islet samples.
(TIF)

S6 Fig RNA-seq analysis against TE database. **A**) Volcano plot showing differentially expressed TEs in *Zfp92* KO vs WT islets collected from 15 weeks old male mice (N = 4). RNA-seq read alignment was done by using random assignment of multi-mapped reads to TE database and differential expression analysis was done globally. The volcano plot only shows differentially expressed TEs. TEs that changed significantly (FDR p_adj_-value <0.1) are shown in red. Select TEs are labeled by the name:family:class. **B**) UCSC Genome Browser view of representative unchanged TEs from top upregulated (RLTR44C) and downregulated (RLTR19B) types of TEs from select locations on chromosomes 3 and 5, respectively (GRCm38/mm10 assembly). Alignments of RNA-seq sequenced reads for the representative WT (blue) and *Zfp92* KO (red) islet samples are displayed by density graphs.
(TIF)

S7 Fig Analysis of ZFP92 binding motif. **A**) Comparison of consensus ZPF92 binding motif (top) predicted by MEME analysis of ZFP92 CUT&RUN peaks with computational predictions of ZFP92 binding site based on the protein sequence at http://zf.princeton.edu/. The sequences from top to bottom are predicted with expanded linear svm, polynomial svm, and B1H algorithms, respectively. Shaded boxes denote similarities with tandem GGAG and GAGG sequences. **B**) Pie charts show the family distribution of LINE, LTR and DNA elements (Fig 9G) containing ZFP92 binding motif.
(TIF)

S8 Fig ZFP92 binding within different loci and its differential effect on LTR TE expression. **A**) UCSC Genome Browser view of *Acacb* locus showing ZFP92 CUT&RUN binding peak and RNA-seq reads for mRNA expression in WT and Zfp92 KO samples (zoomed out view in Fig 11A). Repeat masker and cCRE tracks show ZFP92-bound SINE, LINE, and LTR TEs and nearby distal enhancer. No expression of ZFP92-bound TE elements is detected in Zfp92 KOs. **B**) UCSC Genome Browser view of *Il1r* locus showing ZFP92 CUT&RUN binding peak and RNA-seq reads for mRNA expression in WT and *Zfp92* KO. Repeat masker and cCRE tracks show ZFP92-bound LTR TE and nearby distal enhancer. The expression of the ZFP92-bound LTR element is increased in *Zfp92* KOs (GRCm38/mm10 assembly).
(TIF)

S9 Fig Conservation analysis of ZFP92 proteins. Clastal Omega alignment of ten ZFP92 proteins representing different mammalian branches: 1) dolphin, (ENSTTRP00000003772_Ttru/1-353); 2) cow, *Bos taurus* (ENSBTAP00000059971_Btau/1-411); 3) rabbit, *Oryctolagus cuniculus* (ENSOCUP00000026819_ Ocun /1-401); 4) horse, *Equus ferus cabballus* (ENSECAP00000013403_Ecab/1-485); 5) dog, *Canis lupus familiaris* (ENSCAFP00845031774_Clfa/1-539); 6) rat, *Rattus norvegicus* (ENSRNOP00000076403_Rnor/1-491); 7) marmoset, *Callithrix jacchus* (ENSCJAP00000074532_Cjac/1-4 73); 8) pig, *Sus scrofa* (ENSSSCP00 000056138_Sscr/1-443); 9) *Homo sapiens* (ENSP00000462054_Hsap/1-416); 10) mouse, *Mus musculus* (ENSMUSP00 000033740_Mmus/1-48 8). Protein domains (PROSITE) of mouse ZFP92 are shown underneath. The top gray bar over alignment denotes the degree of conservation. Residues with higher than 80% identity are highlighted in yellow. The image was created by using BioRender.
(TIF)

S10 Fig ZFP92 binding within *Mafb* locus. UCSC Genome Browser view of *Mafb* locus showing ZFP92 CUT&RUN binding peaks and RNA-seq reads for mRNA expression in WT and *Zfp92* KO samples (GRCm38/mm10 assembly). The peaks with significant enrichment (highlighted in blue) occupy conserved regions and SINE elements.
(TIF)

S1 Table Oligonucleotides used in the study. The table lists oligonucleotide names, sequences, applications, and PCR product sizes when applicable.
(DOCX)

S2 Table Results of differential expression analysis of RNAseq datasets from wild type and *Zfp92* knockout islets. Differential expression analysis of RNA-seq datasets was done using DEseq2. Expression levels of genes are presented as normalized counts. baseMean: the average of the normalized counts taken over all samples; log2FoldChange: log2 fold change between the compared groups; lfcSE: standard error of the log2FoldChange estimate; stat: Wald statistic; pvalue = Wald test p-value; padj = Benjamini-Hochberg adjusted p-value.
(XLSX)

S3 Table Results of differential expression analysis of transposable elements using RNAseq datasets from wild type and *Zfp800* knockout pancreata. RNA-seq reads were aligned to TE database by using random assignment per chromosome and globally. Differential expression analysis was done by using DEseq2. Tabs correspond to “chromosomes”, “global” and “1Mb± Mafb” (1Mb upstream and downstream of *Mafb* gene) differential expression analysis of TE expression. baseMean: the average of the normalized counts taken over all samples; log2FoldChange: log2 fold change between the compared groups; lfcSE: standard error of the log2FoldChange estimate; stat: Wald statistic; pvalue = Wald test p-value; padj = Benjamini-Hochberg adjusted p-value.
(XLSX)

S4 Table ZFP92-bound peaks and analysis of genomic features within them. The tabs correspond to ‘ZFP92 binding peaks’, ‘TE enrichment in ZFP92 peaks’ and ‘ZFP92 motif occurrence in TE’ analyses. ZFP92 binding peaks were identified from CUR&RUN data using MACS2. The table contains information for the top 500 enriched peaks (chromosome peak boundaries and enrichment statistics), TEs, chromatin regulatory elements (ENCODE cCREs) and gene features identified within peak boundaries. pvalue = dynamic Poisson distribution p-value; qvalue = Benjamini-Hochberg corrected p-value. Lines for the peaks with multiple TEs are replicated. A unique peak ID identifies each peak. TE enrichment in ZFP92 peaks analysis shows the enrichment of different TE and repeat families in ZFP92 peaks in relation to their genome distribution. The analysis is based on RepeatMasker annotations. ZFP92 motif occurrence in TEs analysis was done by using the FIMO tool in the MEME suite. The table contains information on ZFP92 putative recognition motif location within TEs found in ZFP92 binding peaks.
(XLSX)

S1 Data The tabs contain raw data for each figure. (XLSX)

S1 Method Parameters used for genome-wide mapping of transposable elements. (DOCX)
