# Supplementary figures and images for: ZFP92, a KRAB domain zinc finger protein enriched in pancreatic islets, binds to B1/Alu SINE transposable elements and regulates retroelements and genes
Source: PLoS Genet. 2023 May 8;19(5):e1010729. doi: 10.1371/journal.pgen.1010729 (PMC10166502; doi:10.1371/journal.pgen.1010729)

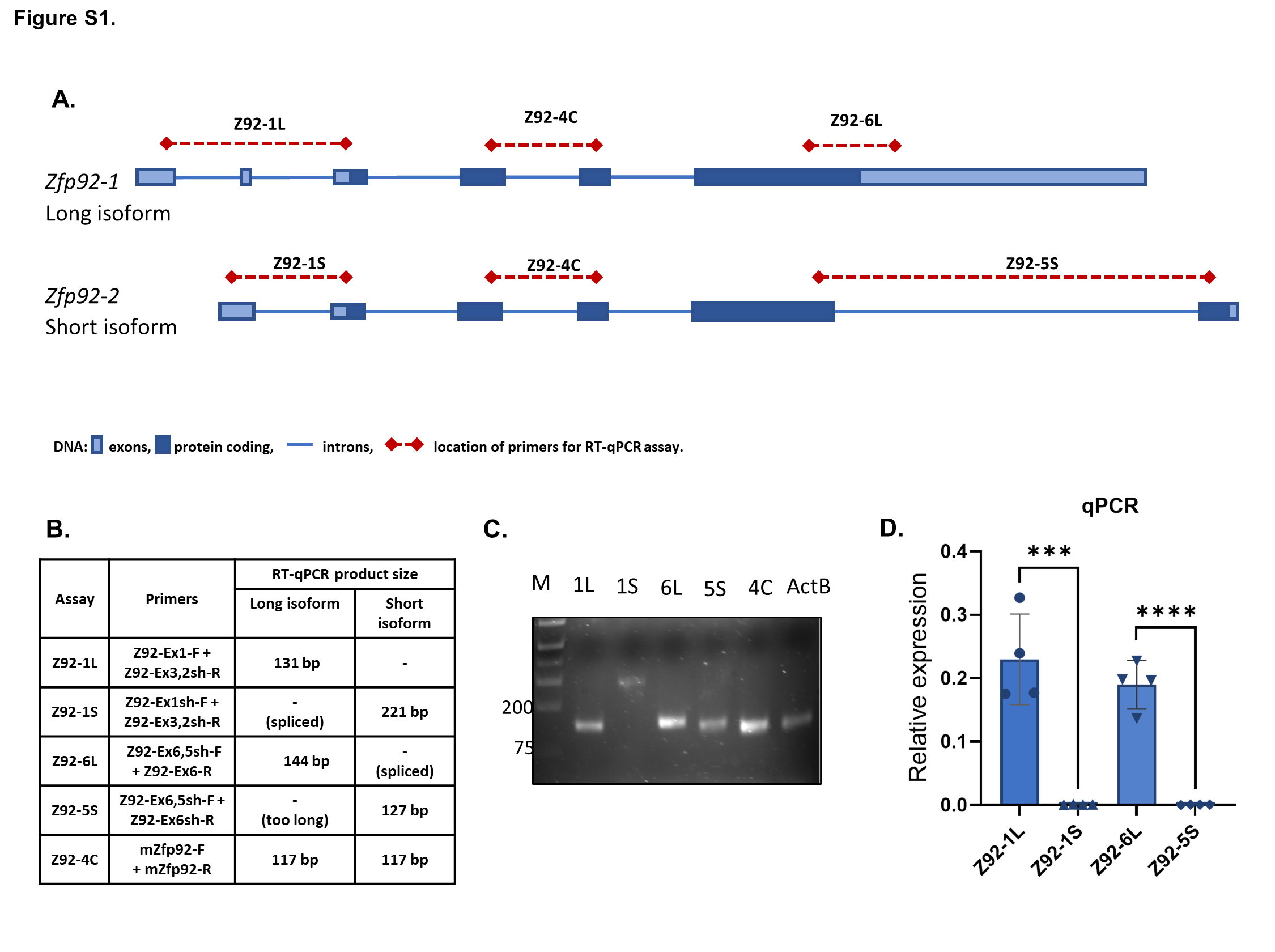

Supplement: S1 Fig — A) Schematic representation of predicted mRNA isoforms 1 and 2 for Zfp92 gene and relative location of primers used for RT-qPCR assays. B) Table listing RT-qPCR assays, primers and expected PCR product sizes for each mRNA form (qPCR cycling protocol). The 4C assay detects both RNA forms. C) Image of the gel showing PCR products for each assay after 40 cycles of qPCR. D) Quantification of relative expression of Zfp92 RNA forms 1 and 2 by RT-qPCR. The expression of individual mRNA forms was normalized to the common 4C assay. Zfp92 mRNA form 1 is predominantly expressed in islets. N = 3, error bars: ± SEM. ***p≤0.001. p-value determined by unpaired t-test. (TIF) [file pgen.1010729.s001.tif]

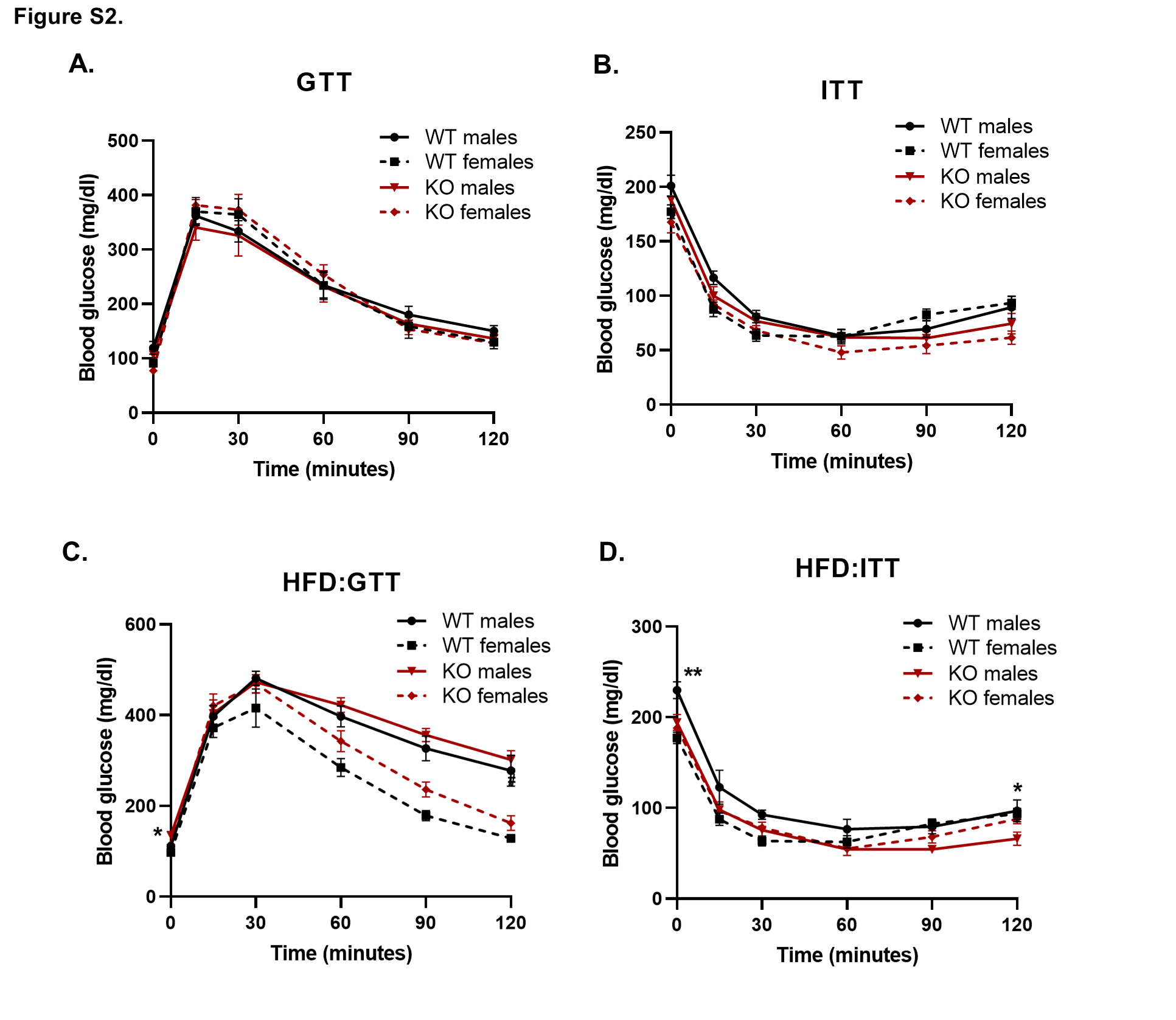

Supplement: S2 Fig — Intraperitoneal glucose tolerance test (GTT) results (A) and insulin tolerance test (ITT) results (B) comparing regular chow-fed wild type (WT) and Zfp92 knockout (KO) male and female mice at 14–15 weeks. No differences in GTT or ITT were observed. N = 7–9 for each sex and genotype. C) GTT results comparing wild type (WT) and Zfp92 knockout (KO) male and female mice at 14–15 weeks after 10 weeks on a high fat diet. N = 8 for each sex and genotype (*p≤0.05: WT vs KO males). D) ITT results comparing wild type (WT) and Zfp92 knockout (KO) male and female mice at 14–15 weeks after 10 weeks on a high fat diet. n = 8 for each sex and genotype (**p≤0.01: WT vs KO males). KO male mice have higher insulin sensitivity than WT mice after 10 weeks on HFD. n = 8 for each sex and genotype. (** p≤0.01; *p≤0.05: WT vs KO males). Error bars: ± SEM. **p≤0.01; *p≤0.05. p-value determined by ANOVA. (TIF) [file pgen.1010729.s002.tif]

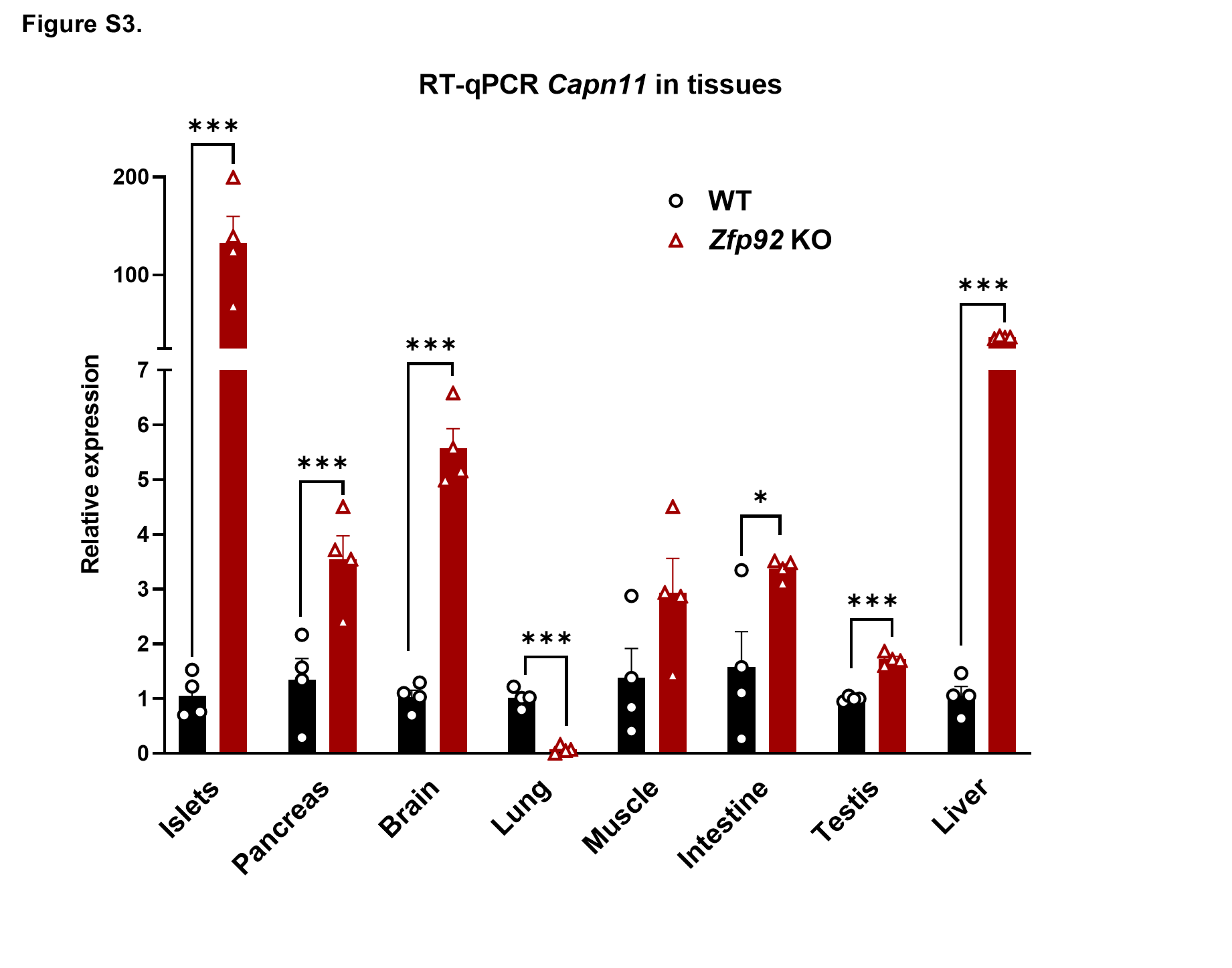

Supplement: S3 Fig — RT-qPCR analysis of expression of IAPez-driven Capn11 transcript (exons 4–5) in Zfp92 KO relative to wild type (WT) mouse tissues. N = 4, error bars: ± SEM. ***p≤0.001; *p≤0.05. p-value is determined by an unpaired t-test. (TIF) [file pgen.1010729.s003.tif]

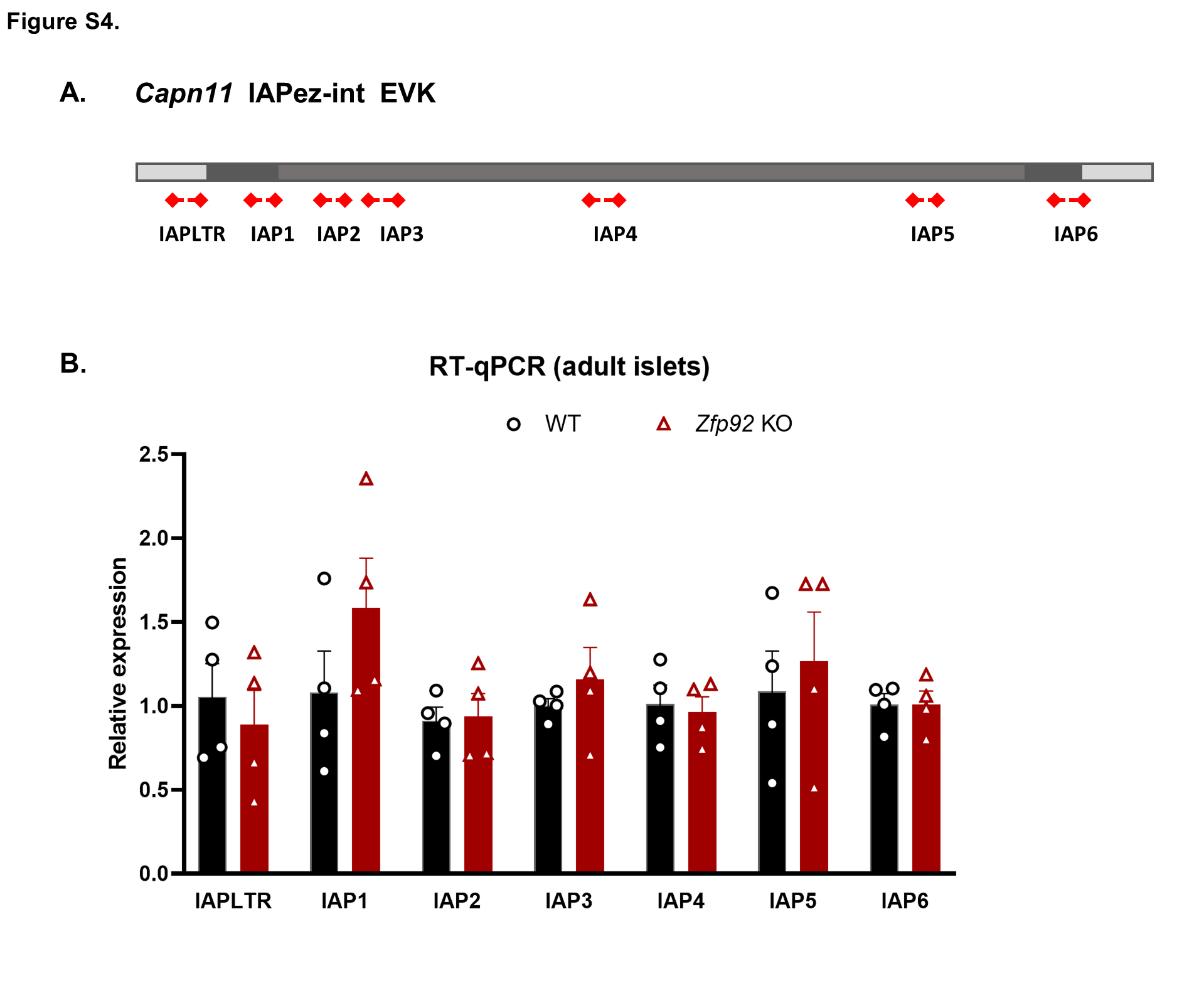

Supplement: S4 Fig — A) Schematic representation of IAPez ERV found in exon 3 of Capn11 gene and relative location of primers used for RT-qPCR assays. LTRs are shown in light gray color, putative ZFP92 binding site is in red. B) RT-qPCR analysis of expression of IAPez with the probes shown in (A) in Zfp92 KO relative to wild type (WT) mouse islets. N = 4, error bars: ± SEM. ***p≤0.001; *p≤0.05. p-value is determined by an unpaired t-test. (TIF) [file pgen.1010729.s004.tif]

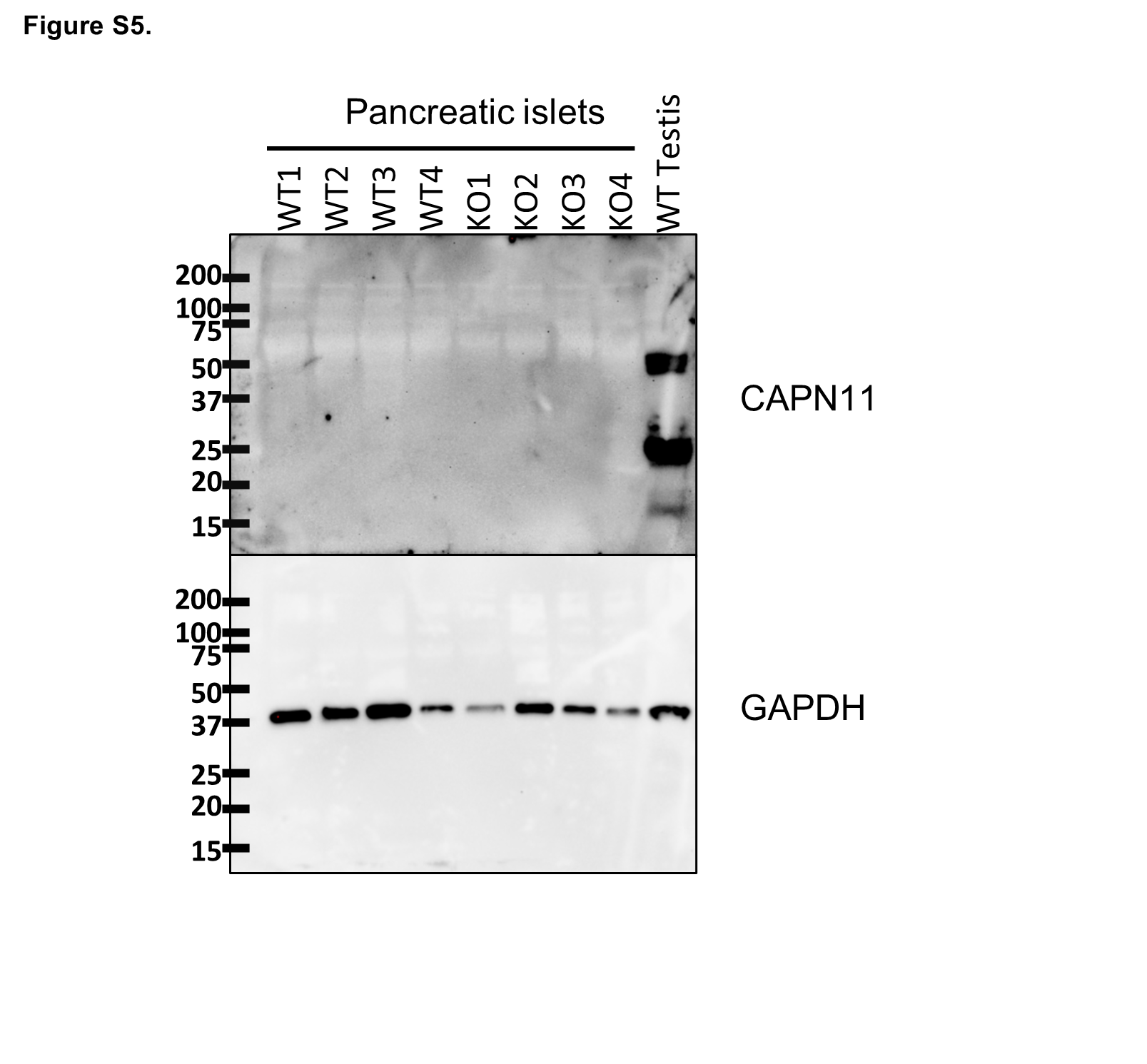

Supplement: S5 Fig — Islet lysates from individual Zfp92 KO and wild type mice were used in Western blot analysis using antibodies raised against the C-terminus of human CAPN11 protein. The testis protein lysate is loaded as a positive control since Capn11 is highly expressed in testis. The predicted full CAPN11 protein size is ~80kD, however, the protein of this is not detected in the testis sample. No specific protein bands are detected in islet samples. (TIF) [file pgen.1010729.s005.tif]

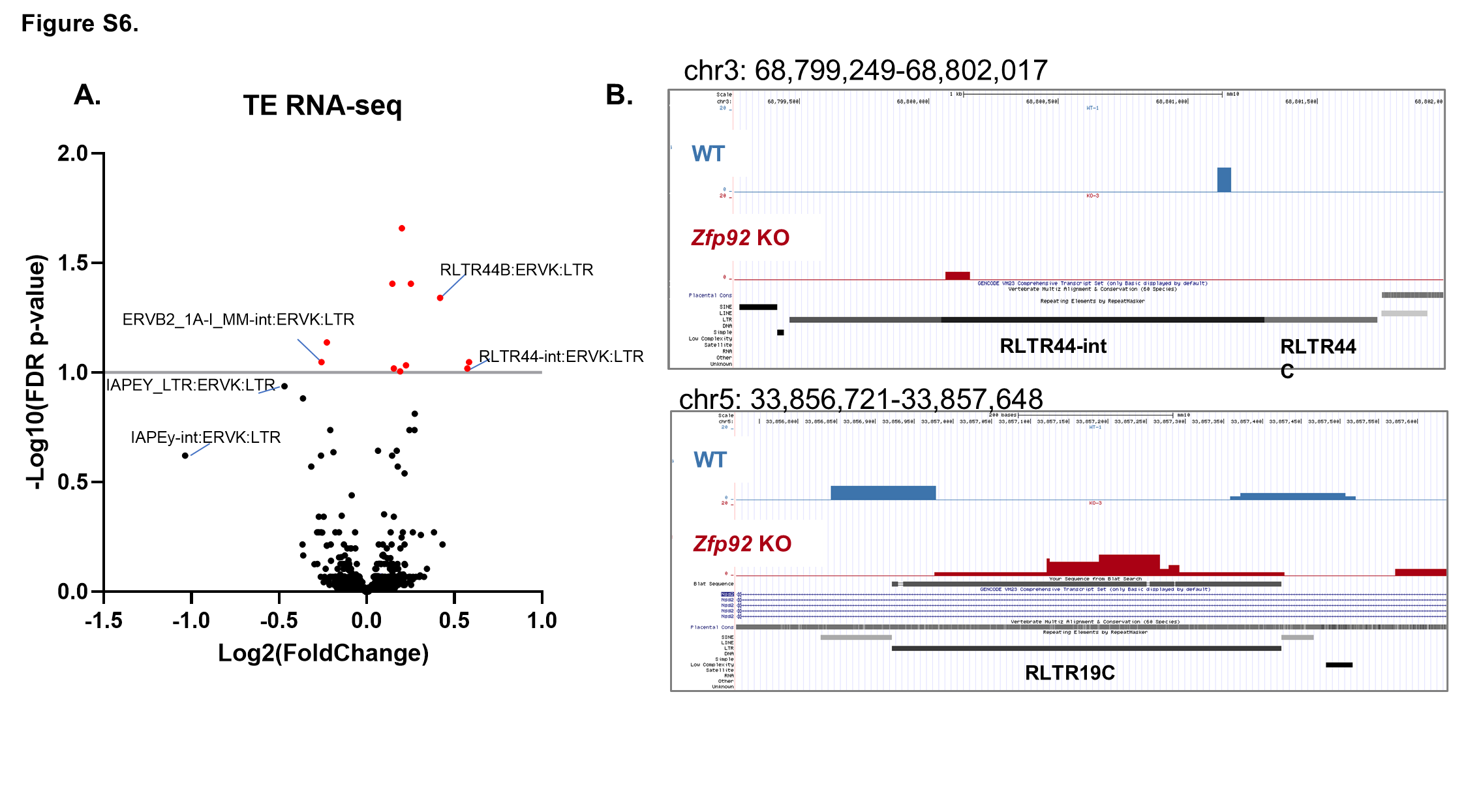

Supplement: S6 Fig — A) Volcano plot showing differentially expressed TEs in Zfp92 KO vs WT islets collected from 15 weeks old male mice (N = 4). RNA-seq read alignment was done by using random assignment of multi-mapped reads to TE database and differential expression analysis was done globally. The volcano plot only shows differentially expressed TEs. TEs that changed significantly (FDR padj-value <0.1) are shown in red. Select TEs are labeled by the name:family:class. B) UCSC Genome Browser view of representative unchanged TEs from top upregulated (RLTR44C) and downregulated (RLTR19B) types of TEs from select locations on chromosomes 3 and 5, respectively (GRCm38/mm10 assembly). Alignments of RNA-seq sequenced reads for the representative WT (blue) and Zfp92 KO (red) islet samples are displayed by density graphs. (TIF) [file pgen.1010729.s006.tif]

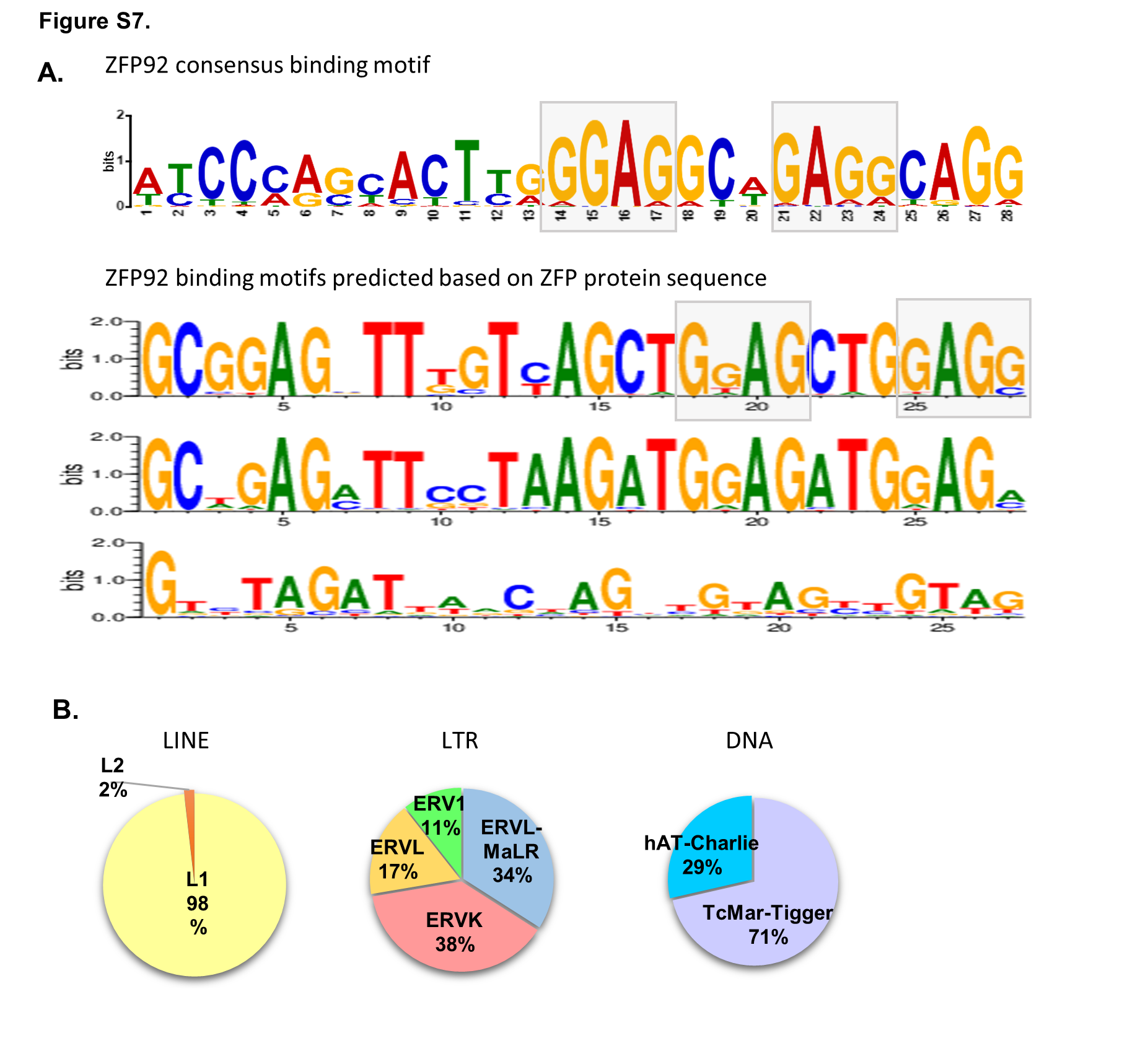

Supplement: S7 Fig — A) Comparison of consensus ZPF92 binding motif (top) predicted by MEME analysis of ZFP92 CUT&RUN peaks with computational predictions of ZFP92 binding site based on the protein sequence at http://zf.princeton.edu/. The sequences from top to bottom are predicted with expanded linear svm, polynomial svm, and B1H algorithms, respectively. Shaded boxes denote similarities with tandem GGAG and GAGG sequences. B) Pie charts show the family distribution of LINE, LTR and DNA elements (Fig 9G) containing ZFP92 binding motif. (TIF) [file pgen.1010729.s007.tif]

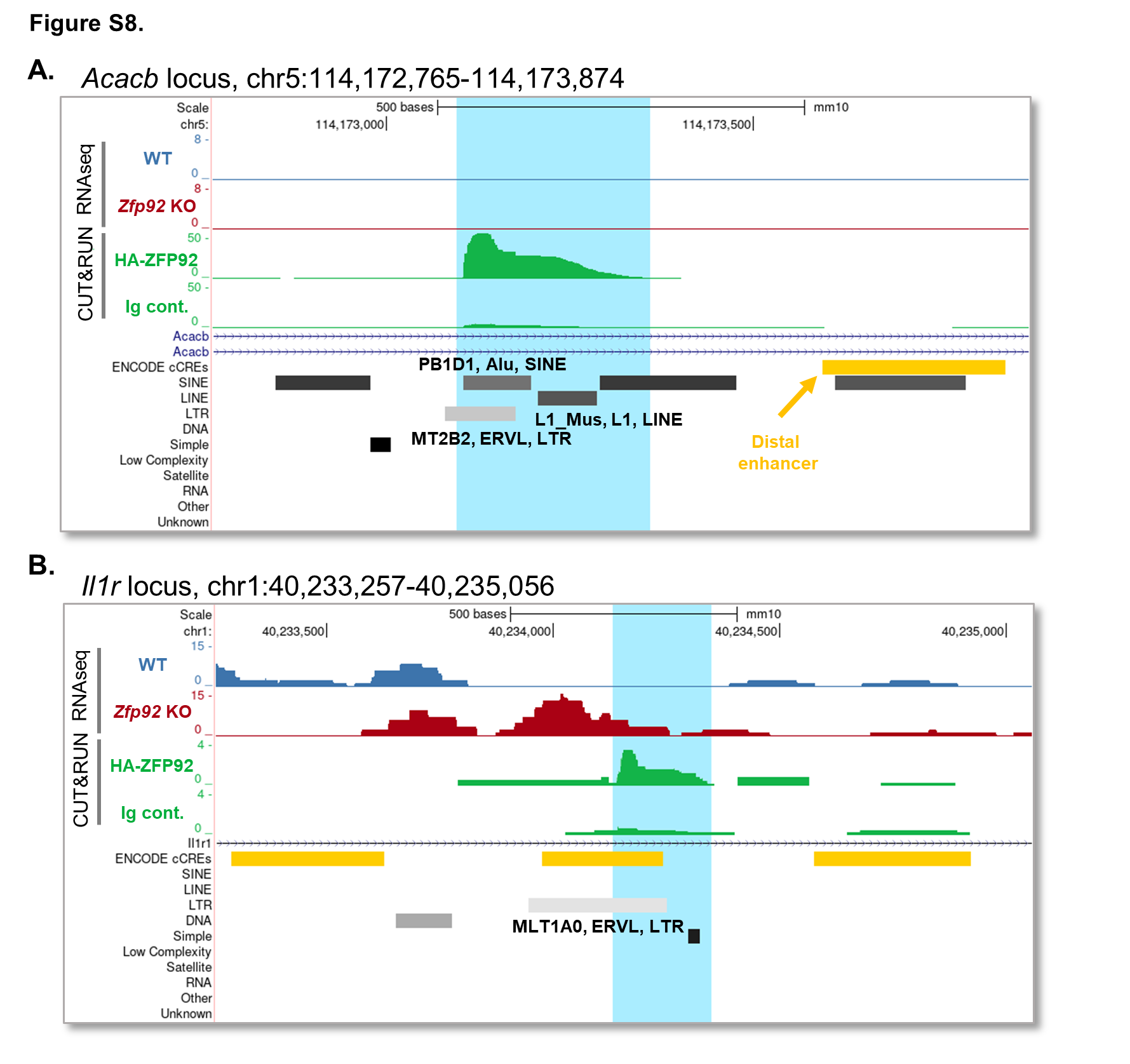

Supplement: S8 Fig — A) UCSC Genome Browser view of Acacb locus showing ZFP92 CUT&RUN binding peak and RNA-seq reads for mRNA expression in WT and Zfp92 KO samples (zoomed out view in Fig 11A). Repeat masker and cCRE tracks show ZFP92-bound SINE, LINE, and LTR TEs and nearby distal enhancer. No expression of ZFP92-bound TE elements is detected in Zfp92 KOs. B) UCSC Genome Browser view of Il1r locus showing ZFP92 CUT&RUN binding peak and RNA-seq reads for mRNA expression in WT and Zfp92 KO. Repeat masker and cCRE tracks show ZFP92-bound LTR TE and nearby distal enhancer. The expression of the ZFP92-bound LTR element is increased in Zfp92 KOs (GRCm38/mm10 assembly). (TIF) [file pgen.1010729.s008.tif]

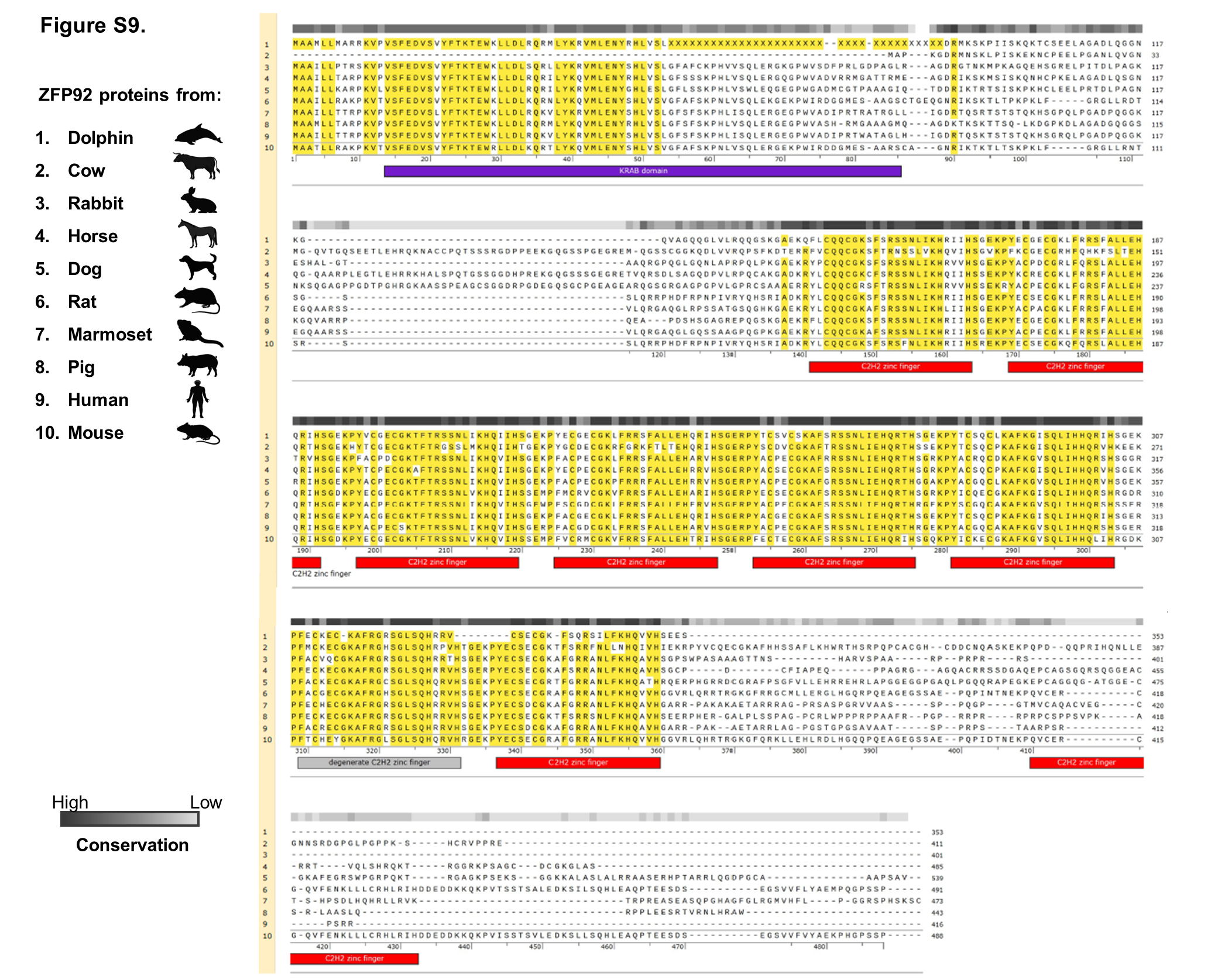

Supplement: S9 Fig — Clastal Omega alignment of ten ZFP92 proteins representing different mammalian branches: 1) dolphin, (ENSTTRP00000003772_Ttru/1-353); 2) cow, Bos taurus (ENSBTAP00000059971_Btau/1-411); 3) rabbit, Oryctolagus cuniculus (ENSOCUP00000026819_ Ocun /1-401); 4) horse, Equus ferus cabballus (ENSECAP00000013403_Ecab/1-485); 5) dog, Canis lupus familiaris (ENSCAFP00845031774_Clfa/1-539); 6) rat, Rattus norvegicus (ENSRNOP00000076403_Rnor/1-491); 7) marmoset, Callithrix jacchus (ENSCJAP00000074532_Cjac/1-4 73); 8) pig, Sus scrofa (ENSSSCP00 000056138_Sscr/1-443); 9) Homo sapiens (ENSP00000462054_Hsap/1-416); 10) mouse, Mus musculus (ENSMUSP00 000033740_Mmus/1-48 8). Protein domains (PROSITE) of mouse ZFP92 are shown underneath. The top gray bar over alignment denotes the degree of conservation. Residues with higher than 80% identity are highlighted in yellow. The image was created by using BioRender. (TIF) [file pgen.1010729.s009.tif]

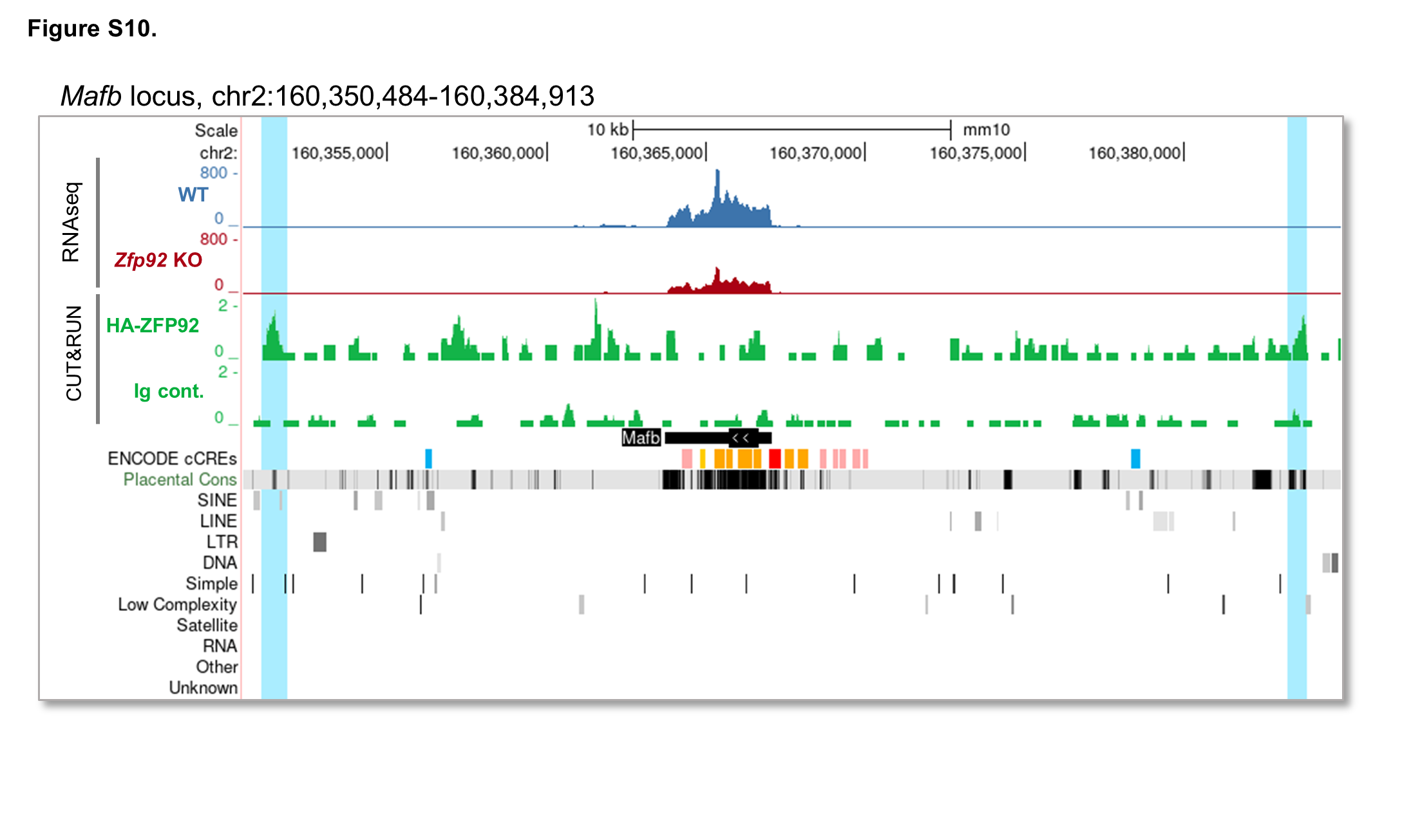

Supplement: S10 Fig — UCSC Genome Browser view of Mafb locus showing ZFP92 CUT&RUN binding peaks and RNA-seq reads for mRNA expression in WT and Zfp92 KO samples (GRCm38/mm10 assembly). The peaks with significant enrichment (highlighted in blue) occupy conserved regions and SINE elements. (TIF) [file pgen.1010729.s010.tif]
