# Supplementary material for: ZFP92, a KRAB domain zinc finger protein enriched in pancreatic islets, binds to B1/Alu SINE transposable elements and regulates retroelements and genes
Source: PLoS Genet. 2023 May 8;19(5):e1010729. doi: 10.1371/journal.pgen.1010729 (PMC10166502; doi:10.1371/journal.pgen.1010729)
Supplement: S1 Table — The table lists oligonucleotide names, sequences, applications, and PCR product sizes when applicable. (DOCX) [file pgen.1010729.s011.docx]

**Table S1. Oligonucleotides used in the study.**

| **Oligo name** | **Sequence (5’ to 3’)** | **PCR product size** | **Application** |
| --- | --- | --- | --- |
| Zfp92crRNA-1  Zfp92 crRNA-2  Zfp92 crRNA-3 | GTTCGCAGCATCCGTGATGA  CAAACGGACCACCATCATCA  CGCAGCATCCGTGATGATGG | Not applicable | CRISPR/Cas9-mediated disruption of *Zfp92* gene |
| KD2Zfp92-Fwd  KD2Zfp92-Rev | AATTCCCGACCACATAACTG    GCAAGAAAGTTCCAAAGCAGAGTC | Targeted: 165 bps  Wild type: 172 bps | Genotyping of *Zfp92^em1Mgn^* mice |
| mZfp92-F  mZfp92-R | CTACAGCCATCTGGTGTCAGTAG  GATCTTGCTGCACTCTCCATTC | 117 bps  (exons 4-5) | RT-qPCR of *Zfp92* |
| Z92-Ex1-F  Z92-Ex3,2sh-R | CCAAAGGTCTCTACGCAGGAC  AGAAGAGTGGCTGCCATAGTC | 131 bps  (exons 1-3) | RT-qPCR of *Zfp92* isoform 1 |
| Z92-Ex1sh-F  Z92-Ex3,2sh-R | GAGCTGAGCAGGAGCTTTCAG  AGACTGTCCAGTGTCTCTGTGAG | 221 bps  (exons 1-2) | RT-qPCR of *Zfp92* isoform 2 |
| Z92-Ex6,5sh-F  Z92Ex6-R | CACGTGCCATGAGTATGGCAAG    ACCTGGTGCTTGAAGAGGTTG | 144 bps  (exon 6) | RT-qPCR of *Zfp92* isoform 1 |
| Z92-Ex6,5sh-F  Z92-Ex6s-R | CACGTGCCATGAGTATGGCAAG    AGTCCAGCACTCCAGAGTTAATG | 127 bps  (exon 5-6) | RT-qPCR of *Zfp92* isoform 2 |
| Ins1-F  Ins1-R | CTATAAAGCTGGTGGGCATCCAG  ACACCAGGTAGAGAGCCTCTAC | 216 bps  (exons 1-2) | RT-qPCR of *Ins1* |
| Gcg-F  Gcg-R | CATTCACCAGCGACTACAGCAA  TCATCAACCACTGCACAAAATCT | 69 bps  (exon 3) | RT-qPCR of *Gcg* |
| Mafb-F  Mafb-R | CCCAGCTTCAGTCCGACTGA  GGTAGTTGCTCGCCATCCA | 67 bps  (exon 1) | RT-qPCR of *Mafb* |
| Capn11-F2  Capn11-R2 | AAACTGCTGTACCGTGTGGTG  GTTCAACCAGTGCCCAAACTG | 99 bps  (exons 4-5) | RT-qPCR of *Capn11* and TE*-Capn11* fusion transcript |
| Capn11-F1    Capn11-R1 | CTTCAGGATGCTGAACTACTCAG  CTCTGCTCGAAGGTCCTCATAG | 151 bps  (exon 1-2) | RT-qPCR of *Capn11* |
| Sox17sRNA-F    Sox17sRNA-R | CGGATTAGGCGAAGGGTTAGGT  TGGTCGTCACTGGCGTATCC | 209 bps  (extended  exon 4) | RT-qPCR of *Sox17* short RNA form  qPCR Fig.10B |
| Sox17lRNA-F    Sox17lRNA-R | CCAACACTCCTCCCAAAGTATCTATC  GAAATGACGATTGAAAACAGTAGC | 134 bps  (exons 1-2) | RT-qPCR of *Sox17* short RNA form |
| Sox17bRNA-F    Sox17bRNA-R | CTTTATGGTGTGGGCCAAAGAC  GCTTCTCTGCCAAGGTCAACG | 122 bps  (exons 4-5) | RT-qPCR of *Sox17* both RNA forms |
| Acacb-F    Acacb-R | CCAGGAGGCTGCATTGAACAC  GTGAAATCTCTGTGCAGGTCCAG | 129 bps  (exons 1-2) | RT-qPCR of *Acacb* |
| Actb-F    Actb-R | GATGCTCCCCGGGCTGCATTC  CTCTTGCTCTGGGCCTCGTCAC | 112 bps  (exons 2-3) | RT-qPCR of *Actb* |
| Hprt-F  Hprt-R | TACGAGGAGTCCTGTTGATGTTGC  GGGACGCAGCAACTGACATTTCTA | 131 bps  (exon 9) | RT-qPCR of *Hprt* |
| IALTR-F  IALTR-R | GTGATGCGCCCTAGGCAATG  GTTATTCGTCGCGTTCTCACGAC | 184 bps | RT-qPCR of IAPez,  IAPLTR region Fig.S4  qPCR Fig.9E |
| IAP-F  IAP-R | CTCATCAGCTGGGGAACGAC  GTACTCTCGTTCCCCAGCTG | 105 bps | RT-qPCR of IAPez,  IAP1 region Fig.S4  qPCR Fig.9E |
| IAP-D2  IAP-R2 | CGTCACTAGATGAGCTCAGGAAG  GCTGGTAATGGGCTGCTTCTTC | 104 bps | RT-qPCR of IAPez,  IAP2 region Fig.S4 |
| IAP3-F    IAP3-R | TGGCGAAAGTCAGCGTACTG  GAATGAATGAGTCTGCGCACTG | 221 bps | RT-qPCR of IAPez,  IAP3 region Fig.S4 |
| IAP4-F    IAP4-R | GAATGGGCCATTGTTGTGGATC  CTGCCAAGGTTTGAACCTGTG | 179 bps | RT-qPCR of IAPez,  IAP4 region Fig.S4 |
| IAP5-F    IAP5-R | CAGACAGCCTTGGTTCTGTCTG  CCAGCATGAGACCTCTCTGTATCT | 130 bps | RT-qPCR of IAPez,  IAP5 region Fig.S4 |
| IAP6-F    IAP6-R | GAAAGGCTCTGCGGCATATATGAG  AGATAGAGGTCAAATCCCCAGTGTG | 198 bps | RT-qPCR of IAPez,  IAP5 region Fig.S4 |
| B1SINE-F  B1SINE-R | GATGCTCCCCGGGCTGCATTC  CTCTTGCTCTGGGCCTCGTCAC | 155 bps | qPCR of B1 SINE |
| TECapn11-F  TECapn11-R | GAACTCAAATATCCACCTGCCTCTG  CACCACACGGTACAGCAGTTTG | 637 bps | qPCR of Capn11-TE spanning region |
| Capn11-5’-R1  Capn11-5’-R2 | GATTACGCCAAGCTTCTCCAGCAGGGCGCTCCAGAAATC  GATTACGCCAAGCTTGATGGGAGGCGTGCACGAATACCAG | Not applicable  (exon 5) | Nested reverse primers for  5’ RACE of *Capn11* |
