# Supplementary material for: ZFP92, a KRAB domain zinc finger protein enriched in pancreatic islets, binds to B1/Alu SINE transposable elements and regulates retroelements and genes
Source: PLoS Genet. 2023 May 8;19(5):e1010729. doi: 10.1371/journal.pgen.1010729 (PMC10166502; doi:10.1371/journal.pgen.1010729)
Supplement: S1 Method — (DOCX) [file pgen.1010729.s016.docx]

**Supplemental method.**

For genome-wide mapping of transposable elements, we optimized the STAR parameters to 1) include not more than 5000 multiple alignments allowed per read, 2) return a single output alignment per multimapper, 3) allow a maximum of 3 mismatchers per read pair, 4) return multiple alignments for each read in random order and also randomizes the choice of the primary alignment from the highest scoring alignments, 5) allow up to 5000 loci anchors to be mapped to, 6) set an upper limit of 350nt as a maximum distance between mates, 7) set the search start point through the read at 30, thereby splitting reads into approximately five pieces, 8) consider only 30000 alignments per read, 9) set 30000 as the maximum number of windows per read, 10) set 399 as the maximum of transcripts per window, 11) set the maximum number of seeds per read at 3000, 12) set the maximum number of seeds per window at 300, 13) and set the maximum number of one seed loci per window at 1000. The parameter string used was:

--outSAMtype BAM SortedByCoordinate --runMode alignReads --outFilterMultimapNmax 5000 --outSAMmultNmax 1 --outFilterMismatchNmax 3 --outMultimapperOrder Random --winAnchorMultimapNmax 5000 --alignEndsType EndToEnd --alignIntronMax 1 --alignMatesGapMax 350 --seedSearchStartLmax 30 --alignTranscriptsPerReadNmax 30000 --alignWindowsPerReadNmax 30000 --alignTranscriptsPerWindowNmax 300 --seedPerReadNmax 3000 --seedPerWindowNmax 300 --seedNoneLociPerWindow 1000
